# Supplementary material for: Statistical inferences for polarity identification in natural language
Source: PLoS One. 2018 Dec 21;13(12):e0209323. doi: 10.1371/journal.pone.0209323 (PMC6303018; doi:10.1371/journal.pone.0209323)
Supplement: S1 Supplementary Materials — (PDF) [file pone.0209323.s001.pdf]

Statistical inferences for polarity identification in natural language

Contents

A Preprocessing of natural language **2**

B Calculation of abnormal returns **3**

C Comparison to dictionary-based sentiment **4**

D Comparison of polarity expressions from movie reviews and financial filings **6**

E Comparison to non-linear models from machine learning **8**

F List of polarity terms **10**

F.1 Study I: Movie reviews 10

F.2 Study II: Financial filings 19

F.3 Overlapping polarity expressions in movie reviews and financial filings 22

G List of polarity bigrams **23**

G.1 Study I: Movie reviews 23

G.2 Study II: Financial filings 32

Appendix A: Preprocessing of natural language

Consistent with previous research [1], we apply the following preprocessing operation to each corpus:

1. **Cleaning.** Each token is matched against a list of orthographically correct words[1] in order to determine whether it is a valid word and thus discard a small number of abbreviations, acronyms and orthographic errors [2]. In the case of financial filings, we remove additional, non-relevant parts via pattern matching (i. e. contact addresses and HTML/XML formatting).

2. **Stop word removal.** We remove words without a deeper meaning [1], such as *the*, *is* and *of*. For this purpose, we utilize a list of 174 stop words from previous research [3].

3. **Stemming.** Stemming reduces inflected words to their stem, even if this stem is not itself a valid root form [1]. Inflected forms are then grouped together. Here, we utilize Porter's stemming algorithm [4].

4. **Document-term matrix.** Let $x_{d,t}$ denote the frequency of how often a term t occurs in document d. The values of $x_{d,t}$ then form the document-term matrix. Many terms occur only once or a small number of times. Therefore, it is common to remove infrequent terms below a certain threshold (e. g. [2, 5, 6]). In our case, we follow [2] and remove sparse terms that appear in less than 5 % of all documents.

5. **Weighting.** As outlined in the main part of the paper, we weight the raw term frequencies in order to lend greater importance to expressions that are especially prevalent in that narrative. The weighting is obtained via the term frequency-inverse document frequency (tf-idf) approach [7].

[1] The utilized dictionary contains 109,581 words and is available from the MIT website (`http://web.mit.edu/moforj/Public/wordsEn.txt`), accessed on January 23, 2017.

Appendix B: Calculation of abnormal returns

A common way to study the effects of certain events on stock prices is to use an event study methodology [8, 9]. This procedure results in an abnormal return that measures the impact without confounding influences. As part of this method, one must first predict a nominal return in the absence of the event under study. Afterwards, one calculates the abnormal return as the difference between the actual and the nominal return.

In our paper, we follow common practices and estimate the nominal return via the market model approach [8]. This model assumes a stable linear relation between the market return $R_m(t)$ and the nominal return $R(t)$ of a stock. The market return $R_m(t)$ stems from a stock market index (in our case the NYSE Composite) along with an estimation window T that comprises the 10 trading days prior to the event.

Appendix C: Comparison to dictionary-based sentiment

We now compare the predictive performance of our method with common dictionaries from previous research. Consistent with the many applications in behavioral research, the dictionaries are used to compute a sentiment score at document level as follows: we compute the difference between the frequency of positive and negative words, normalized by the total number of words. In the case of SentiWordNet, we utilize the individual weights of the words labeled as positive or negative. We then sum over the products of term frequency and SentiWordNet weight.

The results are presented in Table 1. As shown, our method outperforms the dictionaries with regard to both movie reviews and financial disclosures. In the case of movie reviews, the best performing dictionary achieves a mean squared error of 0.1294, which is 90.66 % higher than for the LASSO (mean squared error of 0.0679). We observe a similar pattern for the financial disclosures. Here, the best performing dictionary yields a mean squared error with stock market returns of 16.2511, which is 0.39 % higher than for the LASSO (mean squared error of 16.1980).

We also see large differences between domain-specific and all-purpose dictionaries. For instance, the common Harvard IV dictionary is designed as a general psychological dictionary independent of a particular domain. As a result, we identify instances in relation to the financial disclosures where domain-specific dictionaries obtain superior results. This especially holds true for the Henry and Loughran-McDonald dictionaries, which are both finance-specific. In contrast, these two finance-specific dictionaries lead to inferior performance when applied to the movie reviews.

Table 1 also includes a comparison to the method proposed by [10]. This approach counts frequencies of words from the Loughran-McDonald dictionary. These are then inserted into an ordinary least squares estimator. As a result, it succeeds in assigning words to a small subset of words. However, this approach yields an inferior performance. This pattern also holds true when weighting word occurrences based on term frequency-inverse document frequencies (tf-idf) instead of pure term frequencies.

By misclassifying and erroneously excluding words that are statistically relevant, dictionaries fail to cover the multi-faceted bandwidth of natural language. Altogether, their results reinforce our previous findings that "expert" dictionaries deviate from true perception, for both general and domain-specific audiences.

Finally, we compare our method to a simple OLS estimator instead of the LASSO. The resulting approach lacks variables selection and thus utilizes all words as regressors, while facing statistical risks from multicollinearity and overfitting. Hence, it is not surprising that the OLS estimator yields inferior results in this predictive setting.

Table 1. Comparison to dictionary-based sentiment.

Method	Mean Squared Error	Root Mean Squared Error	Relative Mean Squared Error	Standard Error	Out-of-Sample R^2
STUDY I: MOVIE REVIEWS					
Static Dictionaries					
Harvard IV	0.1294	0.3597	1.9066	0.0578	0.0195
Henry	0.1530	0.3911	2.2540	**0.0126**	−0.1590
Loughran-McDonald	0.1630	0.4036	2.4006	0.0248	−0.2344
QDAP	0.1317	0.3629	1.9407	0.0464	0.0021
SentiWordNet	0.1440	0.3794	2.1209	0.0145	−0.0905
Regression Approaches					
Jegadeesh and Wu (2013) [10] with tf-idf	0.0807	0.2839	1.1885	0.2608	0.3891
Jegadeesh and Wu (2013) [10]	0.0784	0.2798	1.1551	0.2653	0.4061
OLS	0.0804	0.2828	1.1842	0.3168	0.3921
Our approach	**0.0679**	**0.2600**	**1.0000**	0.2531	**0.4864**
STUDY II: FINANCIAL FILINGS					
Static Dictionaries					
Harvard IV	16.2612	4.0238	1.0039	0.0423	−0.0005
Henry	16.2511	4.0225	1.0033	0.0128	0.0002
Loughran-McDonald	16.2539	4.0228	1.0034	0.0188	0.0000
QDAP	16.2532	4.0228	1.0034	0.0300	0.0000
SentiWordNet	16.2546	4.0230	1.0035	**0.0119**	−0.0001
Regression Approaches					
OLS	16.6048	4.0662	1.0251	0.7870	−0.0218
Jegadeesh and Wu (2013) [10] with tf-idf	16.3019	4.0287	1.0064	0.4756	−0.0029
Jegadeesh and Wu (2013) [10]	16.4922	4.0523	1.0182	0.5761	−0.0148
Our approach	**16.1980**	**4.0158**	**1.0000**	0.2320	**0.0035**

Notes: This table compares the sentiment scores from manually selected dictionaries and our statistical procedure in a predictive setting. All results are average over an outer 10-fold cross-validation.

Appendix D: Comparison of polarity expressions from movie reviews and financial filings

A comparison between both the financial filings and movie reviews reveals a clear disparity in the extracted terms, for which we provide statistical evidence in the following: only 38 out of 172 selected words (i. e. 22.09 %) from the financial corpus also appear in the list of extracted words from movie reviews. The agreement predominantly applies to positive words (e. g. *positive* or *strong*) and rarely for negative entries. Table 7 gives an excerpt of the overlapping terms and their coefficients in alphabetical order, while the complete list of overlapping terms is provided in Appendix F. Out of the terms appearing in both results, not all coefficients entail the same polarity. In fact, only 55.26 % actually feature the same direction, while the remaining 44.74 % convey sentiment in the opposite direction. We particularly notice that words expressing uncertainty are often loaded positively in reviews, while having a negative influence in the financial domain. Prominent examples thereof include *although* or *however*.

In addition, we test the correlation between the two lists of estimated coefficients. The correlation amounts to only 0.0216, which is not statistically significant at common significance levels (t-value of 0.5645). As a consequence, this suggests that the reception and interpretation of language is highly domain-specific and each application requires its own tailored dictionary.

Table 2. Polarity Terms That Are Statistically Relevant in Both Movie Reviews and Financial Filings.

	Study I: Movie Reviews			Study II: Financial Filings			
**Word Stem**	**Coef.**	**Positive Doc. (%)**	**Negative Doc. (%)**	**Coef.**	**Positive Doc. (%)**	**Negative Doc. (%)**	**Equal Connotation (i. e. Sign)**
abil	0.0135	0.66	0.34	0.0001	0.49	0.51	✓
actual	−0.0229	0.54	0.46	0.0007	0.49	0.51	✗
allow	0.0148	0.66	0.34	0.0022	0.49	0.51	✓
although	0.0254	0.67	0.33	−0.0036	0.48	0.52	✗
alway	0.0334	0.65	0.35	0.0010	0.50	0.50	✓
appear	−0.0186	0.57	0.43	0.0000	0.49	0.51	✓
becam	0.0016	0.61	0.39	0.0008	0.49	0.51	✓
better	−0.0092	0.55	0.45	0.0007	0.49	0.51	✗
challeng	0.0181	0.68	0.32	−0.0054	0.48	0.52	✗
complex	0.0304	0.78	0.22	−0.0013	0.49	0.51	✗
⋮	⋮	⋮	⋮	⋮	⋮	⋮	⋮

Notes: This table shows an excerpt of terms that were selected by our procedure from both movie reviews and financial disclosures. The word stems are arranged in alphabetical order. In addition, we provide the ratio of documents with a positive or negative label. The symbol "✓" indicates terms that show an equal coefficient sign in both word lists, whereas "✗" indicates a disagreement regarding the word connotation. The full list of all 38 terms with agreement is given in Section F.3.

Table 3 reports further statistical comparisons. Movie reviews contain a higher number of statistically relevant words compared to financial filings. They also entail a higher ratio of positive words, whereas we observe a higher share of negative words in the case of financial filings. As elaborated upon earlier, we observe a higher goodness-of-fit for movie reviews. Similarly, the correlation between the estimated values from the linear model with the gold standard stands at 0.7897 for movie reviews and 0.1110 for financial filings. Finally, we also see that our approach diminishes the problem of multicollinearity among predictors.

Table 3. Summary Statistics of Statistical Inferences.

	Study I: Movie Reviews	Study II: Financial Filings
Regressors before regularization	1195.0000	1724.0000
Extracted terms	549.0000	172.0000
Ratio of extracted terms	45.9414 %	9.9768 %
Positive terms	294.0000	82.0000
Negative terms	255.0000	90.0000
Ratio positive terms	53.5519 %	47.6744 %
Ratio negative terms	46.4480 %	52.3256 %
Coefficients: Mean	−0.0002	0.0001
Coefficients: Min.	−0.1124	−0.0204
Coefficients: 25 % Quantile	−0.0083	−0.0016
Coefficients: Median	0.0008	−0.0001
Coefficients: 75 % Quantile	0.0088	0.0016
Coefficients: Max.	0.0709	0.0325
Coefficients: Std. Dev.	0.0192	0.0049
Coefficients: Skewness	−0.9152	1.3355
Coefficients: Kurtosis	5.2070	12.8537
Adjusted R^2	0.5668	0.0079
Correlation between model estimate and gold standard	0.7897	0.1110
Average VIF before regularization	1.7215	2.1375
Ratio VIF ≥ 4 before regularization	1.5063 %	1.3921 %
Average VIF after regularization	1.2954	1.1812
Ratio VIF ≥ 4 after regularization	0.5464 %	0.0000 %

In addition, Table 3 provides summary statistics of the estimated coefficients, which are likewise visualized by Fig 1 in the form of a kernel density plot. Both distributions are statistically different at the 0.1 % significance level according to a two-sample Kolmogorov-Smirnov test. We note a wider distribution for movie reviews, while that for financial filings features a sharp peak. This is reflected by a standard deviation of 0.0192 for movie reviews compared to 0.0049 in the case of disclosures. Both distributions feature a mean and skewness close to zero, indicating hardly any distortion in either a positive or negative direction. Hence, investors only pay attention to a few, albeit critical, cues in financial statements. This is in contrast to user-generated content where a much richer language helps to convey opinions with explicit and implicit expressions.

Fig 1. Kernel density estimation of word polarity scores from statistical inferences.

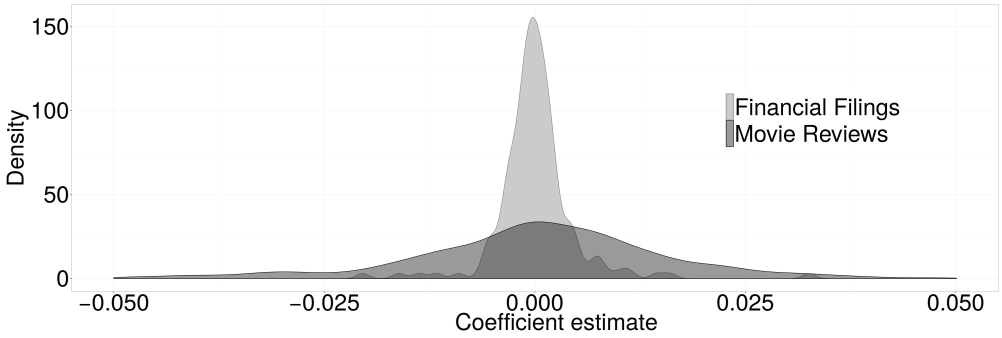

Appendix E: Comparison to non-linear models from machine learning

Since our approach is based on a linear model formulation, we are now interested in potential gains from relaxing the linearity assumption and, instead, use non-linear approaches from machine learning. The latter often features a better fit at the cost of being less interpretable. For this purpose, we implement a predictive setting whereby we compare predictions of user ratings and stock returns at document level. We specifically incorporated the characteristics of our dataset in our choice of models and decided upon the use of random forest, support vector regression and the boosted generalized linear model, since these work well with wide data that entails many regressors in comparison to a fairly small number of observations [11].

We examine the above methods with the same input data as used in our previous studies. Furthermore, we average the predictive performance across 10 different splits into training and test sets in order to ensure the generalizability of our results. It is worth noting that we also experimented with time-preserving splits where the test set incorporates financial disclosures that were released after those from the training set. These yield similar results and are available upon request.

We use the following configuration and parameters when reporting results from machine learning methods. All tuning steps are performed using the `caret` package in the statistical software `R`. If not stated otherwise, we use the default tuning grid from the `caret` package.

- **Support vector regression.** We use a Gaussian radial basis kernel, which is a common choice for text classfication tasks with many predictors (e. g. [12]). The cost parameter C and the kernel parameter σ are tuned using 2-fold cross-validation.

- **Random forest.** We use 500 trees, which is the default choice in that package. The number of randomly selected variables at each split ($mtry$) is tuned using 2-fold cross-validation, such that the overall runtime is still within a feasible range.

- **Boosted generalized linear model.** The number of boosting iterations ($mstop$) is tuned using 2-fold cross-validation with step size $\nu = 0.1$ (which is also a default parameter).

Table 4 reports the results. Machine learning generally yields higher accuracies for movie reviews than for financial disclosures, since it is difficult to predict stock market movements based on news [13]. We also observe a trade-off between predictive performance and interpretability. As expected, machine learning outperforms our linear model for the prediction of both movie reviews and financial disclosures. In the case of movie reviews, the best-performing machine learning model obtains an mean squared error of 0.0620, which is 8.69 % lower than for the LASSO (mean squared error of 0.0679). We also observe a slight improvement with regard to the financial disclosures.

Table 4. Comparison with Prediction Performance of Non-Linear Models.

**Method**	**Mean Squared Error**	**Root Mean Squared Error**	**Standard Error**	**Out-of-Sample R^2**
STUDY I: MOVIE REVIEWS				
Boosted generalized linear model	0.0913<br>(+34.46 %)	0.3022<br>(+15.96 %)	**0.1368**<br>**(−45.95 %)**	0.3083<br>(−36.62 %)
Random forest	0.0810<br>(+19.29 %)	0.2846<br>(+9.22 %)	0.1691<br>(−33.19 %)	0.3863<br>(−20.58 %)
Support vector regression	**0.0620**<br>**(−8.69 %)**	**0.2490**<br>**(−4.44 %)**	0.2465<br>(−2.61 %)	**0.5307**<br>**(+9.11 %)**
Our approach	0.0679	0.2606	0.2531	0.4864
STUDY II: FINANCIAL FILINGS				
Boosted generalized linear model	**16.1939**<br>**(−0.03 %)**	**4.0125**<br>**(−0.01 %)**	**0.1864**<br>**(−19.66 %)**	**0.0038**<br>**(+8.57 %)**
Random forest	16.7352<br>(+3.32 %)	4.0908<br>(+1.87 %)	0.8206<br>(+253.71 %)	−0.0296<br>(−945.71 %)
Support vector regression	16.2219<br>(+0.15 %)	4.0187<br>(+0.07 %)	0.2185<br>(−5.82 %)	0.0021<br>(−40.00 %)
Our approach	16.1980	4.0158	0.2320	0.0035

Notes: This table compares the prediction performance of machine learning approaches and our method. Results originate from averaging the performance across an outer 10-fold cross-validation. The best-performing model in each dimension is highlighted in bold. The relative change compared to our method is shown in parenthesis.

Keeping in mind the value benefits of interpretability, the relative gains in predictive performance from non-linear relationships appear to be rather negligible. As a result, our approach presents a viable alternative that competes well with non-linear models but, at the same time, guarantees full interpretability.

Appendix F: List of polarity terms

In the following section, we report the complete list of word stems that resulted from our statistical procedure of extracting polarity expressions. We also report the following metrics in all subsequent tables.

Standard errors are calculated via the Post-LASSO [14]. The coefficients are generally distributed closely around zero, since the presence of a single word has only a limited effect on the gold standard. In fact, it takes more than one word to elicit considerable changes in the response variable. Hence, we not only report the coefficients, but also utilize several relative metrics that are more convenient in our case. All of this supports us in transforming the coefficients on common scales. For this purpose, we first compute the quantile of each coefficient within the overall distribution of coefficients. This helps us to note the relative polarity of how positively or negatively a word is perceived. Second, we compute the relative magnitude of each coefficient β_i via $\frac{|\beta_i|}{\max |\beta|}$. This compares each term to the impact of the most influential term.

In addition, we provide the relative frequency of how often a term occurs in each corpus. This helps us understand how prevalent a term is within that narrative. For the very same purpose, we also report the inverse document frequency (idf) and, similarly, the ratio of positive and negative documents that it contains.

Finally, the columns show the overlap with common dictionaries. More specifically, these are the Harvard IV psychological dictionary and, in the case of financial disclosures, the finance-specific Loughran-McDonald dictionary, as well. Here, the symbol "$\oplus$" indicates terms that appear in the positive word list and "$\ominus$" in the negative word list of the dictionary in question.

F.1. Study I: Movie reviews

Table 5 reports the extracted terms that convey a positive or negative connotation in movie reviews.

Table 5.: Empirical results of opinionated terms in movie reviews.

Word Stem	Coef. β_i	Relative Magnitude	Quantile	Standard Error	Idf	Relative Freq. (%)	Positive Doc. (%)	Negative Doc. (%)	Harvard IV
abil	0.0135	0.1196	83.03	0.0094	2.4408	8.71	66.06	33.95	$\oplus$
abl	−0.0048	0.0426	31.39	0.0095	1.9253	14.58	57.12	42.88	$\oplus$
absolut	0.0034	0.0299	58.58	0.0097	2.7284	6.53	59.63	40.37	
academi	0.0071	0.0634	69.53	0.0126	2.9089	5.45	79.49	20.51	
accent	−0.0036	0.0316	34.49	0.0095	2.8944	5.53	49.82	50.18	
accept	−0.0105	0.0930	20.99	0.0117	1.5263	21.73	52.39	47.61	$\oplus$
accomplish	0.0043	0.0383	60.95	0.0096	2.7163	6.61	63.44	36.56	$\oplus$
act	−0.0226	0.2007	8.76	0.0098	0.8943	40.89	55.84	44.16	
actor	−0.0113	0.1003	19.17	0.0100	1.0184	36.12	57.63	42.37	
actual	−0.0229	0.2038	8.40	0.0096	1.5117	22.05	53.89	46.11	$\oplus$
affect	0.0116	0.1031	80.11	0.0096	2.5941	7.47	73.80	26.20	$\oplus$
aim	−0.0113	0.1002	19.35	0.0096	2.9387	5.29	44.53	55.47	
alan	−0.0007	0.0063	44.35	0.0094	2.6748	6.89	57.39	42.61	
alien	−0.0016	0.0142	41.25	0.0097	2.9462	5.25	52.47	47.53	$\ominus$
allow	0.0148	0.1319	84.86	0.0096	1.9545	14.16	66.29	33.71	$\oplus$
alon	−0.0047	0.0419	31.94	0.0096	2.4047	9.03	59.29	40.71	
also	0.0200	0.1779	90.33	0.0101	0.7773	45.96	58.50	41.50	
although	0.0254	0.2257	94.53	0.0106	1.1307	32.28	67.26	32.74	
alway	0.0334	0.2968	97.09	0.0095	1.3923	24.85	64.55	35.45	
amaz	0.0059	0.0524	65.88	0.0095	2.7470	6.41	71.03	28.97	$\oplus$
american	0.0028	0.0249	57.49	0.0097	1.6864	18.52	63.75	36.25	
amus	−0.0206	0.1831	9.49	0.0097	2.6049	7.39	45.68	54.32	$\oplus$
angel	−0.0065	0.0576	27.56	0.0094	2.7315	6.51	55.21	44.79	$\oplus$
ann	0.0001	0.0012	47.45	0.0096	2.8314	5.89	59.32	40.68	
anoth	−0.0131	0.1161	16.06	0.0096	0.9788	37.57	58.32	41.68	
answer	0.0085	0.0756	74.28	0.0095	2.2762	10.27	64.98	35.02	

anyon	−0.0205	0.1822	9.68	0.0096	1.7465	17.44	55.10	44.90	
anyth	−0.0301	0.2675	6.21	0.0095	1.4167	24.25	52.64	47.36	
appar	−0.0273	0.2429	7.12	0.0097	2.3384	9.65	51.55	48.45	
appear	−0.0186	0.1651	10.59	0.0097	1.2336	29.13	56.52	43.48	
appreci	0.0033	0.0297	58.40	0.0096	2.7315	6.51	72.39	27.61	$\oplus$
approach	0.0049	0.0433	62.23	0.0097	2.0307	13.12	66.36	33.64	$\oplus$
area	0.0247	0.2198	94.17	0.0096	2.9693	5.13	64.98	35.02	
arm	0.0045	0.0396	61.68	0.0094	2.9126	5.43	58.82	41.18	
art	−0.0023	0.0204	37.41	0.0098	2.0554	12.80	58.50	41.50	$\oplus$
aspect	0.0072	0.0637	69.71	0.0108	2.1049	12.19	70.66	29.34	
attempt	−0.0322	0.2868	5.11	0.0099	1.4118	24.37	52.95	47.05	
attent	−0.0011	0.0100	43.62	0.0096	2.1693	11.43	59.62	40.38	$\oplus$
attract	−0.0031	0.0280	35.95	0.0098	1.9461	14.28	54.97	45.04	$\oplus$
audienc	−0.0147	0.1310	14.06	0.0098	1.0245	35.90	56.98	43.02	
award	0.0307	0.2731	96.54	0.0132	2.3078	9.95	74.50	25.50	$\oplus$
away	0.0068	0.0604	67.89	0.0096	1.3195	26.73	58.07	41.93	
awkward	−0.0159	0.1413	13.51	0.0096	2.9890	5.03	57.54	42.46	$\ominus$
babi	−0.0138	0.1229	15.52	0.0096	2.9424	5.27	52.65	47.35	
bad	−0.1124	1.0000	0.01	0.0103	1.0643	34.50	47.60	52.40	$\ominus$
bar	−0.0040	0.0356	33.76	0.0095	2.7883	6.15	55.84	44.16	$\ominus$
battl	0.0108	0.0965	78.65	0.0101	2.5047	8.17	61.12	38.88	$\ominus$
beat	−0.0003	0.0030	45.81	0.0097	2.7043	6.69	51.04	48.96	$\ominus$
becam	0.0016	0.0138	52.74	0.0096	2.7102	6.65	60.66	39.34	
bed	−0.0043	0.0386	32.85	0.0096	2.7284	6.53	58.41	41.59	
begin	0.0060	0.0533	66.25	0.0097	1.1847	30.58	61.66	38.34	
behind	0.0057	0.0509	65.15	0.0095	2.1675	11.45	60.91	39.09	
believ	−0.0032	0.0280	35.77	0.0096	1.2565	28.47	57.89	42.11	
best	0.0571	0.5077	99.46	0.0098	0.7516	47.16	64.59	35.41	$\oplus$
better	−0.0092	0.0815	23.55	0.0098	1.0942	33.48	55.49	44.51	$\oplus$
bill	−0.0033	0.0289	35.41	0.0095	2.2287	10.77	54.55	45.45	
bit	0.0044	0.0392	61.32	0.0099	1.6515	19.18	60.73	39.27	$\ominus$
bizarr	−0.0007	0.0062	44.53	0.0096	2.8554	5.75	56.25	43.75	$\ominus$
bore	−0.0483	0.4295	2.01	0.0097	2.4998	8.21	37.23	62.77	$\ominus$
boy	−0.0026	0.0232	37.23	0.0097	1.7328	17.68	55.93	44.07	
break	0.0084	0.0745	73.73	0.0095	1.9673	13.98	62.00	38.00	$\ominus$
brilliant	0.0480	0.4268	98.91	0.0095	2.6323	7.19	73.06	26.94	$\oplus$
bring	0.0020	0.0176	55.11	0.0096	1.5300	21.65	59.32	40.68	
bruce	−0.0084	0.0744	24.82	0.0095	2.8416	5.83	54.11	45.89	
buddi	0.0076	0.0673	70.63	0.0096	2.7532	6.37	57.68	42.32	
busi	0.0215	0.1909	91.79	0.0096	1.9774	13.84	59.31	40.69	
buy	−0.0018	0.0157	40.15	0.0095	2.7102	6.65	51.95	48.05	$\oplus$
came	−0.0047	0.0421	31.57	0.0095	2.4136	8.95	54.02	45.98	
camera	0.0110	0.0978	79.02	0.0098	1.9702	13.94	65.90	34.10	
captur	0.0216	0.1918	92.16	0.0097	2.2305	10.75	67.84	32.16	$\ominus$
care	−0.0102	0.0908	21.54	0.0096	1.3740	25.31	57.22	42.78	$\oplus$
career	0.0095	0.0847	76.46	0.0096	2.2030	11.05	62.57	37.43	
carri	−0.0110	0.0980	20.08	0.0094	2.3018	10.01	56.09	43.91	
caus	−0.0067	0.0592	27.19	0.0095	1.9919	13.64	59.44	40.56	
centuri	−0.0054	0.0483	29.93	0.0099	2.4181	8.91	58.52	41.48	
certain	0.0182	0.1623	88.87	0.0096	1.3076	27.05	63.81	36.19	
challeng	0.0181	0.1612	88.51	0.0096	2.6185	7.29	68.49	31.51	$\ominus$
chang	0.0052	0.0464	62.96	0.0096	1.5791	20.62	63.28	36.72	
charm	0.0000	0.0004	46.72	0.0097	2.1248	11.95	65.38	34.62	$\oplus$
cheap	−0.0130	0.1152	16.25	0.0096	2.9424	5.27	49.24	50.76	$\ominus$
children	0.0020	0.0175	54.93	0.0100	2.0491	12.88	61.71	38.29	
choic	0.0019	0.0165	54.38	0.0095	2.2436	10.61	64.97	35.03	
christoph	−0.0068	0.0605	27.01	0.0095	2.7948	6.11	53.59	46.41	
cinema	0.0021	0.0182	55.66	0.0101	2.5145	8.09	71.60	28.40	
cinemat	−0.0091	0.0806	23.73	0.0096	2.5677	7.67	60.16	39.84	
cinematograph	−0.0538	0.4785	1.28	0.0151	1.5524	21.17	44.81	55.19	
class	0.0137	0.1222	83.40	0.0098	2.5395	7.89	61.01	38.99	
classic	0.0152	0.1351	86.32	0.0096	2.1215	11.99	66.33	33.67	$\oplus$
clich	−0.0335	0.2976	4.38	0.0096	2.8520	5.77	46.02	53.98	
close	−0.0010	0.0086	43.80	0.0095	1.6973	18.32	59.87	40.13	$\oplus$
collect	−0.0116	0.1031	18.07	0.0095	2.7470	6.41	49.84	50.16	
combin	0.0062	0.0547	66.79	0.0095	2.7532	6.37	67.40	32.60	
comfort	0.0077	0.0687	71.36	0.0094	2.8113	6.01	62.79	37.21	$\oplus$

common	0.0139	0.1236	83.76	0.0095	2.6894	6.79	67.65	32.35	⊕
compani	0.0158	0.1409	87.96	0.0096	2.5219	8.03	59.95	40.05	⊕
compel	−0.0099	0.0877	21.72	0.0097	2.2048	11.03	62.50	37.50	⊖
complex	0.0304	0.2701	96.17	0.0097	2.5195	8.05	78.41	21.59	⊖
concern	0.0008	0.0073	50.00	0.0096	2.0338	13.08	61.22	38.78	⊖
confess	0.0055	0.0485	63.69	0.0095	2.9732	5.11	58.98	41.02	⊖
confid	0.0230	0.2045	92.71	0.0095	2.9311	5.33	77.53	22.47	⊕
conflict	−0.0021	0.0189	38.14	0.0098	2.8213	5.95	61.07	38.93	⊖
confront	0.0059	0.0528	66.06	0.0096	2.6295	7.21	62.33	37.67	⊖
confus	−0.0109	0.0966	20.44	0.0095	2.4430	8.69	56.78	43.22	⊖
contain	0.0006	0.0050	48.91	0.0095	2.7981	6.09	62.62	37.38	
contrast	0.0130	0.1156	82.30	0.0095	2.8451	5.81	72.51	27.49	
contriv	−0.0207	0.1841	9.13	0.0097	2.9311	5.33	47.57	52.43	
convent	0.0017	0.0150	53.29	0.0095	2.8800	5.61	62.99	37.01	
convinc	−0.0113	0.1009	18.44	0.0095	2.0679	12.64	55.77	44.23	⊕
cop	−0.0021	0.0189	38.33	0.0100	2.4181	8.91	48.43	51.57	
copyright	−0.0168	0.1497	11.87	0.0108	2.9424	5.27	70.45	29.55	
cours	−0.0297	0.2640	6.39	0.0097	1.7510	17.36	53.39	46.61	
craft	0.0015	0.0131	52.01	0.0096	2.8980	5.51	71.74	28.26	
creat	0.0008	0.0067	49.64	0.0097	1.6683	18.86	61.55	38.45	⊕
crime	0.0004	0.0033	48.18	0.0099	2.3199	9.83	56.91	43.09	⊖
critic	0.0015	0.0131	51.83	0.0097	2.0919	12.35	62.14	37.86	⊖
cut	0.0066	0.0591	67.34	0.0097	1.9475	14.26	61.90	38.10	⊖
danc	−0.0017	0.0148	40.52	0.0096	2.4901	8.29	59.28	40.72	⊕
danger	−0.0020	0.0174	39.42	0.0095	2.2531	10.51	56.46	43.54	⊖
dead	−0.0161	0.1429	13.14	0.0096	1.7038	18.20	50.38	49.62	⊖
deal	0.0059	0.0522	65.70	0.0096	1.9616	14.06	63.21	36.79	⊕
debut	0.0125	0.1113	81.57	0.0096	2.8213	5.95	70.13	29.87	
decad	0.0154	0.1368	86.87	0.0095	2.9016	5.49	72.73	27.27	⊖
decid	−0.0218	0.1943	8.95	0.0095	1.5733	20.74	55.30	44.70	
decis	−0.0064	0.0571	27.74	0.0096	2.8944	5.53	56.32	43.68	
delight	0.0515	0.4585	99.09	0.0098	2.2361	10.69	76.82	23.18	⊕
deliv	−0.0023	0.0202	37.60	0.0096	2.0806	12.49	60.80	39.20	
demand	0.0120	0.1069	80.84	0.0096	2.5497	7.81	67.26	32.74	
denni	−0.0468	0.4164	2.19	0.0236	1.4553	23.33	42.21	57.79	
depress	−0.0016	0.0141	41.43	0.0097	2.9654	5.15	51.94	48.06	⊖
deserv	0.0000	0.0001	46.54	0.0096	2.5600	7.73	64.86	35.14	⊕
detail	0.0078	0.0697	72.09	0.0120	1.7138	18.02	62.42	37.58	
determin	0.0083	0.0740	73.36	0.0095	2.7563	6.35	64.47	35.53	
develop	0.0018	0.0159	54.02	0.0098	1.5618	20.97	61.90	38.10	
dialog	−0.0049	0.0435	31.03	0.0101	2.7284	6.53	52.60	47.40	
differ	0.0083	0.0742	73.55	0.0097	1.3899	24.91	64.96	35.04	⊖
direct	−0.0172	0.1529	11.14	0.0105	1.0201	36.06	56.90	43.10	
director	−0.0030	0.0268	36.32	0.0104	0.2691	76.41	57.62	42.38	
disappoint	−0.0080	0.0715	25.37	0.0096	2.2231	10.83	57.56	42.44	⊖
discuss	0.0153	0.1357	86.50	0.0096	2.9424	5.27	74.62	25.38	⊕
distributor	0.0055	0.0487	64.06	0.0128	1.8514	15.70	69.59	30.41	
dog	−0.0113	0.1001	19.53	0.0095	2.4925	8.27	52.66	47.34	
done	−0.0014	0.0128	42.16	0.0096	1.7850	16.78	55.48	44.52	
door	0.0010	0.0088	50.37	0.0095	2.7722	6.25	61.02	38.98	
drama	0.0058	0.0517	65.52	0.0098	1.9253	14.58	63.84	36.16	
draw	0.0148	0.1314	84.49	0.0094	2.6548	7.03	65.06	34.94	
dream	0.0017	0.0151	53.47	0.0096	2.2762	10.27	63.42	36.58	
drop	−0.0021	0.0183	38.69	0.0095	2.7722	6.25	51.44	48.56	⊖
dull	−0.0483	0.4299	1.83	0.0096	2.9126	5.43	32.72	67.28	⊖
earli	0.0125	0.1113	81.76	0.0095	2.0476	12.90	64.09	35.91	
earn	−0.0014	0.0127	42.52	0.0095	2.9577	5.19	62.69	37.31	
easi	0.0353	0.3135	97.82	0.0095	1.8668	15.46	70.67	29.33	⊕
easili	0.0057	0.0505	64.60	0.0095	2.1816	11.29	66.02	33.98	
editor	−0.0400	0.3562	3.47	0.0127	1.8388	15.90	41.46	58.54	
edward	0.0080	0.0709	72.82	0.0094	2.8765	5.63	64.18	35.82	
effect	0.0087	0.0775	74.64	0.0099	1.2849	27.67	66.14	33.86	⊕
element	0.0117	0.1039	80.30	0.0098	2.0353	13.06	70.18	29.82	
encount	0.0146	0.1300	84.31	0.0096	2.8314	5.89	67.12	32.88	
enjoy	0.0172	0.1526	88.14	0.0117	1.1959	30.24	61.49	38.51	⊕
entertain	0.0057	0.0509	65.33	0.0100	1.7171	17.96	58.73	41.27	⊕
equal	0.0115	0.1025	79.93	0.0095	2.3342	9.69	70.93	29.07	⊕

escap	0.0013	0.0114	50.92	0.0099	2.2762	10.27	56.23	43.77	
especi	0.0149	0.1323	85.22	0.0097	1.6630	18.96	65.02	34.98	
exact	−0.0053	0.0473	30.48	0.0096	2.0066	13.44	56.32	43.69	$\oplus$
exampl	−0.0075	0.0666	25.92	0.0095	1.9861	13.72	61.14	38.86	
excel	0.0572	0.5091	99.64	0.0156	1.6258	19.68	64.57	35.43	$\oplus$
excit	0.0076	0.0680	70.99	0.0096	2.8348	5.87	55.10	44.90	$\oplus$
explain	−0.0035	0.0313	34.68	0.0096	2.0156	13.32	57.72	42.28	
express	0.0127	0.1134	82.12	0.0139	1.5590	21.03	62.20	37.80	
extrem	0.0042	0.0378	60.59	0.0097	2.2030	11.05	63.65	36.35	
face	0.0260	0.2317	94.90	0.0096	1.5967	20.26	63.81	36.19	
fail	−0.0318	0.2830	5.30	0.0097	2.0096	13.40	46.80	53.20	$\ominus$
fair	−0.0084	0.0750	24.46	0.0096	2.1231	11.97	53.26	46.74	$\oplus$
fall	−0.0114	0.1016	18.25	0.0097	1.4893	22.55	54.83	45.17	$\ominus$
famili	0.0091	0.0806	75.37	0.0101	1.3398	26.19	62.85	37.15	
famous	0.0013	0.0115	51.10	0.0097	2.6267	7.23	62.98	37.02	$\oplus$
fan	−0.0254	0.2257	8.03	0.0096	2.5022	8.19	54.63	45.37	
far	−0.0127	0.1127	16.61	0.0098	1.5647	20.91	59.50	40.50	
fascin	0.0346	0.3081	97.45	0.0099	2.2455	10.59	77.74	22.26	$\oplus$
fast	0.0221	0.1969	92.52	0.0098	2.3593	9.45	68.29	31.71	
favorit	0.0085	0.0755	74.09	0.0100	2.1248	11.95	65.55	34.45	$\oplus$
fear	0.0151	0.1346	86.14	0.0096	2.3446	9.59	65.63	34.38	$\ominus$
featur	−0.0097	0.0862	21.90	0.0100	1.4683	23.03	58.72	41.28	
feel	0.0036	0.0324	59.49	0.0097	0.9046	40.47	59.82	40.18	
felt	−0.0162	0.1442	12.96	0.0095	2.7102	6.65	52.55	47.45	
femal	−0.0041	0.0369	33.58	0.0099	2.4781	8.39	59.76	40.24	
fiction	0.0321	0.2853	96.90	0.0097	2.5294	7.97	64.66	35.34	
film	−0.0405	0.3603	3.11	0.0112	0.0327	96.78	57.21	42.79	
fine	0.0126	0.1123	81.94	0.0105	1.3560	25.77	68.99	31.01	$\oplus$
fire	−0.0058	0.0514	29.02	0.0096	2.2569	10.47	55.92	44.08	$\ominus$
first	0.0231	0.2058	93.07	0.0097	0.6923	50.04	61.60	38.40	
fit	0.0080	0.0716	73.00	0.0094	2.4640	8.51	62.68	37.32	$\oplus$
flashback	−0.0074	0.0660	26.28	0.0098	2.7851	6.17	56.31	43.69	
flat	−0.0526	0.4677	1.46	0.0096	2.7627	6.31	35.44	64.56	
focus	0.0073	0.0645	70.08	0.0098	2.1282	11.91	71.48	28.52	
follow	0.0083	0.0736	73.18	0.0097	1.4982	22.35	60.14	39.86	
forc	0.0205	0.1827	90.70	0.0097	1.4691	23.01	61.46	38.54	$\ominus$
form	0.0015	0.0134	52.38	0.0095	2.0968	12.29	65.20	34.80	
former	−0.0043	0.0380	33.22	0.0096	2.3614	9.43	52.75	47.25	
formula	−0.0094	0.0837	23.00	0.0098	2.7627	6.31	44.62	55.38	
found	−0.0054	0.0481	30.11	0.0096	1.6494	19.22	52.18	47.82	
frank	0.0136	0.1213	83.22	0.0095	2.3018	10.01	57.09	42.91	$\oplus$
fresh	0.0069	0.0611	68.25	0.0095	2.7013	6.71	67.26	32.74	$\oplus$
frighten	0.0093	0.0828	75.73	0.0095	2.6691	6.93	65.42	34.58	$\ominus$
frustrat	−0.0023	0.0200	37.78	0.0095	2.9274	5.35	63.06	36.94	$\ominus$
full	0.0078	0.0692	71.90	0.0096	1.7305	17.72	63.70	36.30	$\oplus$
fun	0.0150	0.1333	85.77	0.0099	1.8502	15.72	58.70	41.30	$\oplus$
funni	−0.0097	0.0860	22.09	0.0106	1.5281	21.69	55.80	44.20	$\oplus$
futur	0.0087	0.0777	74.82	0.0095	2.6351	7.17	64.90	35.10	
gag	−0.0200	0.1775	9.86	0.0102	2.8080	6.03	39.74	60.26	
genr	0.0071	0.0630	69.35	0.0100	2.4974	8.23	57.28	42.72	
genuin	−0.0097	0.0860	22.27	0.0096	2.2474	10.57	59.74	40.26	$\oplus$
get	−0.0290	0.2583	6.57	0.0106	0.3267	72.13	54.20	45.80	$\ominus$
give	−0.0016	0.0144	40.88	0.0100	0.6308	53.22	57.66	42.34	$\oplus$
goe	−0.0005	0.0047	45.26	0.0098	1.2322	29.17	55.55	44.45	
good	−0.0012	0.0107	43.25	0.0098	0.6876	50.28	56.46	43.54	$\oplus$
got	−0.0037	0.0332	34.31	0.0096	1.7685	17.06	52.69	47.31	
great	0.0709	0.6306	100.00	0.0098	1.1526	31.58	66.29	33.71	$\oplus$
green	−0.0030	0.0266	36.50	0.0094	2.8872	5.57	54.12	45.88	
ground	−0.0016	0.0144	41.06	0.0095	2.6295	7.21	59.56	40.44	
group	−0.0165	0.1470	12.60	0.0096	1.9050	14.88	57.58	42.42	
guess	−0.0120	0.1072	17.34	0.0097	2.4091	8.99	50.67	49.33	
guy	0.0155	0.1376	87.05	0.0100	1.5235	21.79	54.45	45.55	
hair	−0.0047	0.0414	32.30	0.0097	2.7658	6.29	57.46	42.54	
half	−0.0058	0.0518	28.84	0.0096	1.9948	13.60	55.95	44.05	
head	−0.0044	0.0388	32.67	0.0096	1.4744	22.89	55.85	44.15	
heart	0.0068	0.0606	68.07	0.0096	1.7994	16.54	68.84	31.16	$\oplus$
heavi	−0.0113	0.1005	18.80	0.0095	2.6049	7.39	53.51	46.49	

help	0.0005	0.0045	48.73	0.0098	1.4101	24.41	54.09	45.91	⊕
henri	0.0158	0.1406	87.78	0.0094	2.8765	5.63	63.12	36.88	
hero	−0.0088	0.0780	23.91	0.0097	2.1265	11.93	55.78	44.22	⊕
hide	−0.0008	0.0069	44.17	0.0096	2.8348	5.87	58.16	41.84	
high	0.0057	0.0508	64.79	0.0108	1.3803	25.15	61.16	38.84	
hilari	0.0373	0.3314	98.00	0.0096	2.6633	6.97	75.64	24.36	⊕
hire	0.0035	0.0310	58.95	0.0097	2.8014	6.07	50.99	49.01	
histori	0.0035	0.0308	58.76	0.0098	2.4271	8.83	64.25	35.75	
honest	0.0240	0.2133	93.98	0.0095	2.9539	5.21	70.88	29.12	⊕
hope	−0.0171	0.1522	11.32	0.0096	1.7339	17.66	52.26	47.74	⊕
horror	−0.0026	0.0234	37.05	0.0100	2.6185	7.29	55.34	44.66	⊖
howev	0.0210	0.1868	91.43	0.0106	1.1375	32.06	64.61	35.39	
human	0.0056	0.0500	64.42	0.0098	1.6349	19.50	65.68	34.32	⊕
idea	−0.0316	0.2810	5.48	0.0096	1.7362	17.62	50.68	49.32	
imag	0.0077	0.0681	71.17	0.0099	1.9475	14.26	67.37	32.63	
imagin	0.0018	0.0163	54.20	0.0096	2.1816	11.29	61.42	38.58	⊕
immedi	0.0039	0.0347	59.86	0.0095	2.6130	7.33	58.86	41.14	
impact	0.0048	0.0430	61.87	0.0096	2.8944	5.53	71.12	28.88	
import	0.0230	0.2047	92.89	0.0096	1.9645	14.02	68.95	31.05	⊕
imposs	0.0017	0.0153	53.65	0.0095	2.7532	6.37	61.76	38.24	
includ	0.0019	0.0169	54.57	0.0098	1.2240	29.40	61.14	38.86	
incred	0.0001	0.0008	47.09	0.0096	2.9693	5.13	67.70	32.30	⊖
individu	0.0103	0.0913	77.38	0.0098	2.4998	8.21	69.34	30.66	⊕
initi	0.0149	0.1329	85.59	0.0095	2.8179	5.97	71.24	28.76	
innoc	0.0282	0.2505	95.44	0.0095	2.3038	9.99	64.40	35.60	⊕
instead	−0.0409	0.3638	2.92	0.0095	1.6146	19.90	51.41	48.59	
intellig	0.0060	0.0536	66.43	0.0097	2.1049	12.19	64.59	35.41	⊕
intens	0.0469	0.4167	98.73	0.0097	2.3785	9.27	74.35	25.65	
intent	−0.0018	0.0163	39.79	0.0095	2.6103	7.35	58.42	41.58	
interest	−0.0332	0.2956	4.57	0.0098	1.0648	34.48	53.82	46.18	⊕
intrigu	0.0057	0.0508	64.97	0.0098	2.5096	8.13	69.78	30.22	⊕
job	0.0037	0.0326	59.68	0.0098	1.4545	23.35	59.02	40.98	
joke	−0.0021	0.0183	38.87	0.0106	2.2512	10.53	47.63	52.37	⊕
joy	0.0141	0.1256	84.13	0.0095	2.9654	5.15	75.58	24.42	⊕
just	−0.0096	0.0850	22.63	0.0102	0.4475	63.92	56.13	43.88	⊕
keep	0.0002	0.0022	48.00	0.0095	1.1456	31.80	60.18	39.82	
key	0.0104	0.0923	77.74	0.0095	2.4047	9.03	64.60	35.40	
kid	−0.0081	0.0724	25.19	0.0107	1.3353	26.31	56.19	43.81	⊕
kill	−0.0055	0.0492	29.57	0.0105	1.4700	22.99	50.91	49.09	⊖
killer	−0.0015	0.0133	41.79	0.0100	2.4114	8.97	50.11	49.89	⊖
kind	0.0035	0.0312	59.13	0.0107	0.9987	36.84	58.57	41.43	⊕
kiss	0.0013	0.0119	51.46	0.0095	2.8314	5.89	61.36	38.64	⊕
know	0.0044	0.0388	61.14	0.0097	0.9160	40.01	57.61	42.39	⊕
lack	−0.0450	0.4001	2.38	0.0097	1.8030	16.48	48.61	51.39	⊖
leader	0.0200	0.1781	90.52	0.0096	2.8247	5.93	61.28	38.72	
learn	−0.0087	0.0776	24.09	0.0096	1.6329	19.54	58.28	41.72	⊕
least	−0.0672	0.5974	0.92	0.0097	1.4840	22.67	47.58	52.42	
left	−0.0009	0.0083	43.98	0.0096	1.6126	19.94	56.81	43.19	
level	−0.0012	0.0103	43.44	0.0097	2.0081	13.42	62.35	37.65	
lie	0.0023	0.0208	56.57	0.0096	2.2531	10.51	62.17	37.83	⊖
life	0.0551	0.4900	99.28	0.0102	0.6967	49.82	63.79	36.21	
light	−0.0084	0.0745	24.64	0.0095	2.0037	13.48	61.63	38.37	⊕
like	−0.0331	0.2940	4.75	0.0104	0.2124	80.86	56.92	43.08	⊕
limit	0.0071	0.0628	69.17	0.0099	2.1165	12.05	68.66	31.34	⊖
listen	0.0078	0.0692	71.72	0.0096	2.7376	6.47	60.49	39.51	
local	0.0079	0.0699	72.27	0.0096	1.9475	14.26	63.03	36.97	
long	−0.0367	0.3265	3.84	0.0099	0.9987	36.84	55.64	44.36	
loos	−0.0140	0.1242	15.33	0.0095	2.8348	5.87	54.08	45.92	
love	0.0109	0.0965	78.84	0.0104	0.7545	47.02	61.05	38.96	⊕
low	−0.0005	0.0045	45.44	0.0097	2.5809	7.57	55.15	44.85	⊖
make	−0.0191	0.1701	10.22	0.0099	0.2937	74.55	57.42	42.58	⊕
maker	−0.0001	0.0013	46.17	0.0100	2.7627	6.31	65.51	34.49	
male	−0.0057	0.0506	29.20	0.0100	2.5420	7.87	60.41	39.59	
man	0.0049	0.0432	62.05	0.0098	0.8028	44.81	61.93	38.07	⊕
manag	−0.0016	0.0146	40.70	0.0096	1.6247	19.70	58.42	41.58	⊕
mani	0.0305	0.2709	96.36	0.0097	0.7734	46.14	63.25	36.75	
mari	0.0069	0.0616	68.98	0.0094	2.4663	8.49	59.29	40.71	

marriag	0.0053	0.0469	63.14	0.0096	2.5420	7.87	68.02	31.98	⊕
marvel	0.0390	0.3472	98.18	0.0096	2.9577	5.19	79.62	20.38	⊕
master	0.0214	0.1903	91.61	0.0096	2.5471	7.83	71.68	28.32	⊕
match	0.0112	0.0998	79.75	0.0095	2.6267	7.23	66.02	33.98	
materi	−0.0325	0.2894	4.93	0.0096	1.9199	14.66	55.31	44.69	
matur	0.0347	0.3087	97.63	0.0102	2.2550	10.49	74.67	25.33	⊕
may	0.0258	0.2297	94.71	0.0098	1.1363	32.10	64.65	35.35	
mayb	−0.0027	0.0244	36.68	0.0095	2.6633	6.97	44.99	55.01	
mean	0.0111	0.0987	79.38	0.0095	1.6360	19.48	62.26	37.74	⊖
memor	0.0030	0.0271	58.03	0.0096	2.5170	8.07	69.55	30.45	⊕
men	0.0045	0.0396	61.50	0.0100	1.5263	21.73	63.14	36.86	
mental	−0.0051	0.0456	30.84	0.0096	2.9654	5.15	54.65	45.35	
mention	−0.0131	0.1163	15.88	0.0096	2.6022	7.41	52.02	47.98	
mere	−0.0048	0.0427	31.21	0.0096	2.5995	7.43	55.91	44.09	
messag	−0.0006	0.0057	44.71	0.0109	1.6177	19.84	58.51	41.49	
might	−0.0242	0.2150	8.22	0.0097	1.0901	33.62	54.07	45.93	
mild	−0.0146	0.1298	14.24	0.0097	2.6662	6.95	48.85	51.15	⊕
mine	0.0193	0.1716	89.06	0.0136	1.8300	16.04	59.78	40.22	⊖
minor	0.0196	0.1743	89.97	0.0094	2.5522	7.79	66.41	33.59	
minut	−0.0392	0.3491	3.65	0.0107	1.2621	28.31	52.79	47.21	
moment	0.0036	0.0322	59.31	0.0101	1.1893	30.44	59.65	40.35	⊕
moral	0.0026	0.0231	57.30	0.0097	2.5888	7.51	60.11	39.89	⊕
most	−0.0142	0.1267	14.79	0.0096	2.8113	6.01	51.50	48.51	
motion	0.0217	0.1933	92.34	0.0107	1.7838	16.80	68.25	31.75	
move	0.0042	0.0375	60.41	0.0096	1.4937	22.45	64.68	35.32	
movi	−0.0671	0.5966	1.10	0.0130	0.1665	84.66	56.06	43.94	
much	−0.0026	0.0235	36.87	0.0097	0.4880	61.39	57.96	42.04	
name	−0.0107	0.0951	20.63	0.0097	0.9443	38.89	58.04	41.96	
natur	0.0090	0.0804	75.19	0.0096	1.6757	18.72	66.60	33.40	⊕
necessari	0.0015	0.0132	52.19	0.0096	2.7658	6.29	60.63	39.37	
need	−0.0060	0.0536	28.47	0.0096	1.2530	28.57	56.36	43.64	⊖
neither	−0.0055	0.0491	29.75	0.0096	2.5782	7.59	55.53	44.47	
nevertheless	0.0012	0.0105	50.73	0.0096	2.2781	10.25	71.15	28.85	
nice	−0.0166	0.1479	12.05	0.0096	2.0171	13.30	51.05	48.95	⊕
none	−0.0152	0.1349	13.87	0.0096	2.3179	9.85	56.80	43.20	
normal	0.0207	0.1845	91.25	0.0095	2.7315	6.51	73.01	26.99	⊕
noth	−0.0018	0.0158	39.97	0.0097	1.2405	28.93	56.35	43.65	
notic	0.0072	0.0640	69.90	0.0094	2.8113	6.01	61.79	38.21	
nuditi	−0.0195	0.1731	10.04	0.0108	1.7874	16.74	61.46	38.54	
observ	0.0093	0.0832	76.10	0.0095	2.8213	5.95	66.44	33.56	
obvious	−0.0112	0.0997	19.71	0.0097	1.8983	14.98	54.93	45.07	
often	0.0134	0.1193	82.85	0.0098	1.7116	18.06	66.59	33.41	
one	0.0023	0.0206	56.21	0.0102	0.0785	92.45	57.24	42.76	
opposit	0.0077	0.0688	71.54	0.0095	2.9890	5.03	68.65	31.35	⊖
oscar	0.0148	0.1315	84.68	0.0095	2.8729	5.65	80.57	19.43	
other	0.0123	0.1091	81.21	0.0095	1.7886	16.72	65.59	34.41	
outsid	0.0020	0.0181	55.48	0.0095	2.4853	8.33	63.55	36.45	⊖
pain	−0.0166	0.1479	12.23	0.0106	2.0399	13.00	60.52	39.48	⊖
parodi	−0.0285	0.2537	6.76	0.0099	2.9500	5.23	45.04	54.96	
part	0.0007	0.0059	49.46	0.0098	0.7939	45.21	58.90	41.10	
particular	−0.0118	0.1050	17.52	0.0099	1.7465	17.44	57.16	42.84	⊕
pass	0.0004	0.0038	48.55	0.0095	2.2417	10.63	54.89	45.11	⊕
patrick	0.0139	0.1235	83.58	0.0094	2.9850	5.05	56.52	43.48	
pay	0.0032	0.0287	58.22	0.0097	2.2436	10.61	58.76	41.24	⊕
perfect	0.0707	0.6292	99.82	0.0096	1.6515	19.18	74.17	25.83	⊕
period	−0.0005	0.0047	45.08	0.0097	2.4974	8.23	64.56	35.44	
perspect	0.0051	0.0450	62.41	0.0099	2.9577	5.19	80.00	20.00	
phone	−0.0103	0.0912	21.36	0.0095	2.9811	5.07	57.48	42.52	
plan	0.0001	0.0013	47.82	0.0099	2.0216	13.24	55.66	44.34	
plot	−0.0308	0.2736	5.66	0.0100	1.1906	30.40	52.37	47.63	⊖
point	−0.0133	0.1180	15.70	0.0096	1.2267	29.32	58.38	41.62	⊖
polit	0.0025	0.0223	57.12	0.0098	2.2380	10.67	67.79	32.21	⊕
poor	−0.0405	0.3602	3.29	0.0129	1.4831	22.69	53.26	46.74	⊖
portray	0.0111	0.0991	79.57	0.0098	2.2531	10.51	70.91	29.09	
posit	0.0013	0.0118	51.28	0.0095	2.3872	9.19	60.00	40.00	⊕
possibl	−0.0060	0.0535	28.65	0.0095	1.8140	16.30	57.97	42.03	
power	0.0300	0.2672	95.81	0.0097	1.6768	18.70	70.51	29.49	

predict	−0.0412	0.3661	2.74	0.0097	2.3219	9.81	44.60	55.40	
premis	−0.0145	0.1294	14.42	0.0100	2.4901	8.29	46.51	53.49	⊕
presenc	−0.0113	0.1006	18.62	0.0097	2.5319	7.95	54.02	45.98	
pretti	−0.0110	0.0977	20.26	0.0096	2.2030	11.05	45.75	54.25	⊕
previous	0.0029	0.0257	57.85	0.0096	2.4047	9.03	63.94	36.06	
privat	0.0106	0.0939	78.11	0.0097	2.9539	5.21	56.71	43.30	
problem	−0.0284	0.2526	6.94	0.0097	1.2551	28.51	51.51	48.49	⊖
process	0.0097	0.0862	76.83	0.0095	2.8554	5.75	65.97	34.03	
produc	0.0015	0.0136	52.56	0.0117	0.9382	39.13	62.69	37.32	
project	−0.0157	0.1397	13.69	0.0095	2.6351	7.17	54.87	45.13	
promis	−0.0178	0.1585	10.77	0.0095	2.2859	10.17	47.94	52.06	⊕
protagonist	0.0001	0.0007	46.90	0.0096	2.5395	7.89	58.99	41.01	
provid	−0.0053	0.0474	30.30	0.0097	1.7534	17.32	59.86	40.14	⊕
pure	0.0151	0.1342	85.95	0.0095	2.8014	6.07	60.53	39.47	⊕
put	−0.0063	0.0563	28.11	0.0095	1.3732	25.33	56.62	43.38	
qualiti	−0.0065	0.0579	27.38	0.0096	2.2703	10.33	55.32	44.68	⊕
quiet	0.0206	0.1834	91.06	0.0095	2.9089	5.45	75.46	24.54	
quit	0.0106	0.0947	78.29	0.0097	1.4134	24.33	64.61	35.39	⊖
rais	0.0079	0.0700	72.45	0.0095	2.6103	7.35	65.22	34.78	⊖
rang	0.0008	0.0067	49.82	0.0095	2.7690	6.27	61.47	38.54	
rare	0.0108	0.0964	78.47	0.0096	1.9602	14.08	66.81	33.19	
rate	−0.0061	0.0544	28.29	0.0142	0.7203	48.66	60.14	39.86	
reach	0.0022	0.0197	56.03	0.0095	2.5145	8.09	67.41	32.59	
real	0.0016	0.0141	53.11	0.0097	1.1615	31.30	60.31	39.69	⊕
realiti	0.0009	0.0078	50.19	0.0096	2.6130	7.33	66.21	33.79	
realli	−0.0017	0.0155	40.33	0.0097	1.2474	28.73	55.01	44.99	
reason	−0.0142	0.1264	14.97	0.0095	1.3599	25.67	54.86	45.14	⊕
recogn	0.0098	0.0870	77.01	0.0095	2.5756	7.61	65.35	34.65	
recommend	0.0393	0.3495	98.36	0.0138	1.6832	18.58	59.78	40.22	
refer	−0.0087	0.0770	24.28	0.0096	2.4949	8.25	58.11	41.89	
reflect	0.0198	0.1758	90.15	0.0168	1.7294	17.74	62.39	37.61	
relationship	0.0056	0.0498	64.24	0.0103	1.4973	22.37	66.25	33.75	
remain	0.0029	0.0256	57.67	0.0096	2.1066	12.17	64.37	35.63	
remark	0.0092	0.0820	75.55	0.0097	2.5995	7.43	69.62	30.38	⊕
rememb	0.0014	0.0127	51.65	0.0096	2.3572	9.47	61.81	38.19	
repres	0.0006	0.0057	49.28	0.0097	2.5471	7.83	69.90	30.10	
reserv	−0.0127	0.1132	16.43	0.0193	1.4902	22.53	43.09	56.91	
rest	−0.0034	0.0302	35.04	0.0096	1.9992	13.54	52.80	47.20	⊕
reveal	0.0133	0.1186	82.67	0.0096	2.4640	8.51	66.90	33.10	
review	−0.0741	0.6594	0.55	0.0169	0.6297	53.28	51.48	48.52	
ridicul	−0.0271	0.2413	7.49	0.0098	2.3425	9.61	42.00	58.00	⊖
rock	0.0043	0.0381	60.77	0.0096	2.7254	6.55	55.18	44.82	
role	0.0149	0.1325	85.41	0.0099	1.0666	34.42	62.39	37.61	
romanc	−0.0046	0.0412	32.49	0.0098	2.0445	12.94	59.26	40.74	⊕
routin	−0.0031	0.0278	36.14	0.0095	2.9500	5.23	45.42	54.58	
said	−0.0021	0.0188	38.51	0.0096	2.0522	12.84	53.19	46.81	
satisfi	0.0194	0.1723	89.24	0.0095	2.5345	7.93	68.01	31.99	⊕
save	−0.0207	0.1840	9.31	0.0095	1.9759	13.86	48.85	51.15	⊕
scale	−0.0357	0.3174	4.20	0.0162	1.6207	19.78	55.76	44.24	
scene	−0.0110	0.0981	19.90	0.0101	0.5194	59.49	57.82	42.18	
school	−0.0141	0.1255	15.15	0.0116	1.8238	16.14	56.56	43.44	
scott	−0.0020	0.0182	39.06	0.0122	1.4991	22.33	54.65	45.35	
screen	−0.0093	0.0827	23.18	0.0103	1.1737	30.92	60.08	39.92	
screenwrit	−0.0257	0.2286	7.85	0.0118	1.5628	20.95	43.09	56.91	
script	−0.0117	0.1038	17.89	0.0102	0.8874	41.17	54.92	45.08	
search	0.0079	0.0703	72.63	0.0096	2.4181	8.91	60.09	39.91	
seem	−0.0364	0.3241	4.02	0.0100	0.6555	51.92	54.67	45.33	
seen	0.0110	0.0978	79.20	0.0097	1.3939	24.81	58.62	41.38	
sequenc	0.0055	0.0487	63.87	0.0100	1.7060	18.16	59.63	40.37	
seri	−0.0006	0.0053	44.90	0.0098	1.8363	15.94	55.39	44.61	
servic	−0.0019	0.0170	39.60	0.0095	2.8908	5.55	53.24	46.76	⊖
sever	0.0067	0.0593	67.52	0.0096	1.6811	18.62	64.16	35.84	⊖
sex	−0.0096	0.0852	22.45	0.0106	1.4426	23.63	58.83	41.17	
shock	−0.0035	0.0307	34.86	0.0097	2.5370	7.91	57.07	42.93	⊖
short	0.0069	0.0615	68.80	0.0098	1.8350	15.96	62.33	37.67	⊖
side	0.0024	0.0212	56.76	0.0096	1.8338	15.98	62.00	38.00	
sign	−0.0124	0.1101	16.98	0.0095	2.6548	7.03	50.57	49.43	

silli	−0.0169	0.1501	11.68	0.0097	2.4901	8.29	46.75	53.25	⊖
simpl	0.0237	0.2105	93.62	0.0098	2.0522	12.84	73.25	26.75	
simpli	−0.0020	0.0178	39.24	0.0100	2.2646	10.39	56.35	43.65	
sinc	0.0018	0.0158	53.84	0.0098	0.9762	37.67	59.28	40.72	
singl	−0.0166	0.1476	12.41	0.0096	2.1099	12.13	54.20	45.80	
sister	−0.0106	0.0944	20.81	0.0097	2.2978	10.05	60.04	39.96	
sleep	−0.0064	0.0569	27.92	0.0096	2.5677	7.67	52.60	47.40	
slight	0.0067	0.0599	67.71	0.0094	2.6407	7.13	61.62	38.38	⊖
slow	−0.0074	0.0662	26.10	0.0096	2.6212	7.27	56.04	43.96	
slowli	0.0120	0.1064	80.66	0.0096	2.9539	5.21	70.88	29.12	
small	0.0182	0.1622	88.69	0.0096	1.5840	20.52	66.02	33.98	
smart	0.0274	0.2441	95.26	0.0096	2.8146	5.99	67.33	32.67	⊕
smoke	0.0006	0.0051	49.09	0.0095	2.9654	5.15	57.36	42.64	
social	0.0087	0.0775	74.46	0.0097	2.6407	7.13	67.23	32.77	
societi	0.0153	0.1358	86.68	0.0096	2.6894	6.79	68.53	31.47	
solid	0.0064	0.0572	67.16	0.0098	2.2646	10.39	70.58	29.42	
somehow	−0.0160	0.1422	13.33	0.0095	2.7851	6.17	53.07	46.93	
someon	−0.0015	0.0137	41.61	0.0098	1.3685	25.45	54.55	45.45	
song	0.0195	0.1738	89.42	0.0098	2.4781	8.39	70.95	29.05	
soon	−0.0191	0.1694	10.41	0.0096	1.7083	18.12	55.24	44.76	
sound	−0.0014	0.0122	43.07	0.0096	1.9489	14.24	56.80	43.20	⊕
speak	0.0196	0.1741	89.79	0.0097	2.1502	11.65	66.21	33.79	
spend	−0.0124	0.1102	16.79	0.0095	1.8578	15.60	56.21	43.79	
spent	−0.0056	0.0501	29.38	0.0095	2.8520	5.77	55.36	44.64	
spot	−0.0022	0.0200	37.96	0.0097	2.6548	7.03	51.14	48.86	⊖
state	0.0076	0.0674	70.81	0.0100	1.6897	18.46	62.99	37.01	⊕
steal	−0.0145	0.1290	14.60	0.0095	2.3363	9.67	53.93	46.07	⊖
stephen	−0.0079	0.0699	25.55	0.0095	2.6605	6.99	53.71	46.29	
stick	−0.0034	0.0300	35.22	0.0094	2.8624	5.71	54.55	45.45	⊖
still	0.0155	0.1376	87.23	0.0095	1.0818	33.90	61.93	38.07	
stone	−0.0103	0.0919	21.17	0.0094	2.9424	5.27	54.17	45.83	
store	0.0266	0.2369	95.08	0.0095	2.7786	6.21	64.95	35.05	
stori	−0.0047	0.0417	32.12	0.0104	0.3004	74.05	59.16	40.84	
strength	0.0117	0.1040	80.48	0.0096	2.8179	5.97	76.92	23.08	
strong	0.0140	0.1245	83.95	0.0099	1.7850	16.78	68.93	31.07	
student	0.0021	0.0189	55.84	0.0104	2.5835	7.55	54.76	45.24	
studi	0.0051	0.0452	62.60	0.0096	2.4226	8.87	70.05	29.96	⊖
studio	0.0096	0.0855	76.65	0.0096	2.7470	6.41	57.63	42.37	
stuff	−0.0042	0.0371	33.40	0.0096	2.8451	5.81	48.45	51.55	
stupid	−0.0271	0.2412	7.67	0.0096	2.8554	5.75	39.24	60.76	⊖
subject	0.0019	0.0169	54.75	0.0121	1.4300	23.93	60.77	39.23	⊖
subtitl	−0.0113	0.1005	18.98	0.0104	2.8980	5.51	57.97	42.03	
subtl	0.0155	0.1377	87.41	0.0095	2.8314	5.89	77.97	22.03	⊕
support	0.0196	0.1741	89.60	0.0097	1.7779	16.90	68.56	31.44	⊕
suppos	−0.0699	0.6215	0.73	0.0097	1.8540	15.66	41.07	58.93	
sure	−0.0003	0.0024	45.99	0.0095	1.7017	18.24	56.19	43.81	
surpris	0.0238	0.2115	93.80	0.0096	1.3811	25.13	63.83	36.17	
suspens	0.0001	0.0013	47.63	0.0098	2.8944	5.53	49.46	50.54	⊖
tale	0.0094	0.0837	76.28	0.0097	1.6619	18.98	65.47	34.53	
talent	−0.0071	0.0628	26.46	0.0097	2.0871	12.41	57.81	42.19	⊕
talk	−0.0043	0.0384	33.03	0.0096	1.5762	20.68	57.00	43.00	
team	−0.0014	0.0124	42.71	0.0097	2.4569	8.57	50.82	49.18	
teenag	−0.0038	0.0342	34.13	0.0102	1.8997	14.96	57.54	42.46	
teenagers	−0.0229	0.2035	8.58	0.0123	2.3098	9.93	57.14	42.86	
ten	0.0000	0.0002	46.36	0.0096	2.6605	6.99	55.71	44.29	
tension	0.0106	0.0939	77.92	0.0098	2.3937	9.13	61.27	38.73	⊖
terribl	−0.0093	0.0823	23.36	0.0095	2.8729	5.65	50.88	49.12	⊖
that	−0.0083	0.0734	25.00	0.0102	1.4242	24.07	56.85	43.15	
thing	−0.0123	0.1095	17.16	0.0098	0.8865	41.21	55.16	44.84	
third	0.0124	0.1101	81.39	0.0094	2.5782	7.59	65.26	34.74	
thoma	0.0020	0.0180	55.30	0.0095	2.7043	6.69	56.42	43.58	
three	0.0093	0.0831	75.92	0.0097	1.4126	24.35	60.87	39.13	
today	0.0148	0.1319	85.04	0.0096	2.5269	7.99	69.25	30.75	
told	0.0060	0.0538	66.61	0.0098	1.9788	13.82	61.71	38.29	
tom	0.0064	0.0570	66.98	0.0094	2.2455	10.59	60.00	40.00	
toni	−0.0169	0.1507	11.50	0.0094	2.8179	5.97	54.85	45.15	
top	0.0010	0.0088	50.55	0.0119	1.6535	19.14	60.02	39.98	

touch	0.0084	0.0749	73.91	0.0096	2.0585	12.76	69.64	30.36	
tough	0.0103	0.0915	77.56	0.0096	2.7254	6.55	61.28	38.72	
track	−0.0014	0.0123	42.89	0.0096	2.6076	7.37	53.93	46.07	
tradit	0.0179	0.1596	88.33	0.0096	2.5756	7.61	72.44	27.56	⊕
tragedi	0.0301	0.2676	95.99	0.0097	2.7073	6.67	74.55	25.45	⊖
treat	0.0075	0.0669	70.44	0.0095	2.2493	10.55	60.04	39.96	⊕
tri	−0.0306	0.2723	5.84	0.0100	0.7335	48.02	52.29	47.71	⊖
true	0.0233	0.2071	93.44	0.0097	1.7430	17.50	67.58	32.42	⊕
truli	0.0016	0.0139	52.92	0.0096	2.6633	6.97	62.46	37.54	
truth	0.0133	0.1182	82.49	0.0095	2.3981	9.09	68.79	31.21	⊕
turn	0.0069	0.0613	68.44	0.0098	0.8028	44.81	58.94	41.06	⊖
typic	−0.0039	0.0347	33.95	0.0096	2.2588	10.45	56.41	43.59	
ultim	0.0041	0.0368	60.04	0.0098	2.2781	10.25	63.74	36.26	
unbear	−0.0273	0.2428	7.30	0.0140	2.8485	5.79	57.59	42.41	⊖
unbearable	0.0308	0.2741	96.72	0.0250	2.3322	9.71	59.26	40.74	
understand	0.0155	0.1382	87.60	0.0097	1.8489	15.74	68.78	31.22	⊕
unexpect	0.0248	0.2202	94.35	0.0096	2.9089	5.45	74.36	25.64	⊖
unfortun	−0.0512	0.4554	1.65	0.0102	1.9574	14.12	43.42	56.58	⊖
uniqu	0.0416	0.3697	98.55	0.0098	2.4781	8.39	73.81	26.19	⊕
univers	−0.0005	0.0044	45.63	0.0102	2.1606	11.53	59.10	40.90	
unlik	0.0001	0.0010	47.27	0.0096	2.3322	9.71	68.93	31.07	
unusu	0.0233	0.2070	93.25	0.0095	2.8554	5.75	73.96	26.04	
valu	−0.0051	0.0456	30.66	0.0096	2.6605	6.99	52.57	47.43	⊕
version	0.0023	0.0207	56.39	0.0097	2.0774	12.53	60.45	39.55	
video	−0.0069	0.0612	26.65	0.0115	2.1198	12.01	54.08	45.92	
view	0.0053	0.0475	63.33	0.0098	1.7534	17.32	65.63	34.37	
violent	−0.0014	0.0128	41.98	0.0098	2.6720	6.91	58.38	41.62	⊖
visual	0.0101	0.0901	77.19	0.0100	2.0679	12.64	64.93	35.07	⊕
war	0.0041	0.0368	60.22	0.0106	1.9687	13.96	62.23	37.77	⊖
wast	−0.0762	0.6776	0.37	0.0144	1.6463	19.28	52.54	47.46	⊖
watch	−0.0069	0.0611	26.83	0.0097	1.1381	32.04	55.67	44.33	
water	−0.0047	0.0421	31.76	0.0094	2.5809	7.57	55.15	44.85	
way	0.0206	0.1832	90.88	0.0095	0.5961	55.09	59.68	40.32	
wealthi	−0.0014	0.0128	42.34	0.0097	2.8451	5.81	49.83	50.17	
week	0.0215	0.1914	91.98	0.0119	2.2324	10.73	61.64	38.36	
well	0.0024	0.0212	56.94	0.0097	0.9938	37.02	62.87	37.13	⊕
wild	−0.0078	0.0695	25.73	0.0095	2.5420	7.87	59.14	40.86	⊖
will	0.0054	0.0480	63.51	0.0100	0.3928	67.52	57.96	42.04	⊕
wish	−0.0174	0.1547	10.95	0.0097	2.3915	9.15	56.11	43.89	
wit	0.0069	0.0614	68.62	0.0097	2.3384	9.65	59.83	40.17	
within	0.0088	0.0784	75.00	0.0096	2.7223	6.57	66.57	33.43	
wonder	0.0292	0.2598	95.63	0.0097	1.4358	23.79	65.91	34.09	⊕
work	0.0074	0.0655	70.26	0.0098	0.5817	55.89	59.72	40.28	
world	0.0123	0.1090	81.03	0.0111	0.8470	42.87	57.13	42.87	
wors	−0.0442	0.3934	2.56	0.0097	2.6435	7.11	38.48	61.52	⊖
worst	−0.1011	0.8992	0.19	0.0132	1.8164	16.26	52.21	47.79	⊖
worth	−0.0117	0.1038	17.71	0.0096	1.8540	15.66	56.12	43.88	⊕
write	0.0051	0.0455	62.78	0.0095	2.1265	11.93	59.30	40.70	
written	−0.0032	0.0287	35.59	0.0134	1.4579	23.27	60.17	39.83	
wrong	−0.0305	0.2716	6.03	0.0095	2.0871	12.41	52.01	47.99	⊖
yet	0.0339	0.3012	97.27	0.0097	1.6768	18.70	66.67	33.33	
york	−0.0164	0.1455	12.78	0.0096	2.2436	10.61	61.39	38.61	
young	0.0004	0.0036	48.36	0.0097	1.1394	32.00	62.80	37.20	
your	−0.0094	0.0838	22.82	0.0097	2.2665	10.37	54.14	45.86	

F.2. Study II: Financial filings

Table 6 reports verbal expressions that convey positive or negative information in financial disclosures (Form 8-K filings).

Table 6.: Empirical results of opinionated terms in financial filings.

Word Stem	Coef. β_i	Relative Magnitude	Quantile	Standard Error	Idf	Relative Freq. (%)	Positive Doc. (%)	Negative Doc. (%)	Harvard IV	Loughran-McDonald
abil	0.0001	0.0038	55.56	0.0042	0.8927	40.96	49.04	50.96	$\oplus$	
actual	0.0007	0.0214	63.16	0.0045	0.4560	63.38	49.09	50.91	$\oplus$	
advertis	−0.0056	0.1719	3.51	0.0042	2.4241	8.86	48.35	51.65		
agenc	0.0006	0.0195	61.99	0.0041	1.4674	23.05	48.73	51.27		
aggreg	−0.0010	0.0318	36.85	0.0046	1.2531	28.56	47.97	52.03	$\oplus$	
agreement	0.0001	0.0024	54.39	0.0039	1.2407	28.92	48.61	51.39	$\oplus$	
allow	0.0022	0.0668	81.29	0.0039	1.1855	30.56	49.02	50.98	$\oplus$	
although	−0.0036	0.1105	9.95	0.0038	1.6852	18.54	48.01	51.99		
alway	0.0010	0.0302	66.67	0.0037	1.4817	22.72	49.51	50.49		
amort	−0.0004	0.0116	46.79	0.0054	1.3073	27.06	48.76	51.24		
amount	−0.0001	0.0022	50.88	0.0052	0.6093	54.37	48.63	51.37		
analysi	−0.0024	0.0723	20.47	0.0037	1.7285	17.75	48.63	51.37		
annum	0.0056	0.1725	93.57	0.0039	2.3783	9.27	48.00	52.00		
anticip	−0.0006	0.0188	41.53	0.0041	0.9762	37.67	49.09	50.91		
appear	0.0000	0.0010	51.47	0.0038	1.9044	14.89	48.85	51.15		
asset	0.0005	0.0157	59.65	0.0042	0.5972	55.03	48.90	51.10	$\oplus$	
assumpt	−0.0007	0.0220	40.36	0.0042	1.0180	36.13	48.67	51.33		
automot	0.0035	0.1073	87.14	0.0037	2.9020	5.49	49.72	50.28		
avoid	0.0051	0.1577	92.99	0.0037	2.1581	11.55	48.82	51.18	$\ominus$	
becam	0.0008	0.0242	64.92	0.0037	2.1777	11.33	49.16	50.84		
better	0.0007	0.0219	63.75	0.0040	1.9438	14.32	49.21	50.79	$\oplus$	$\oplus$
billion	0.0018	0.0544	76.61	0.0041	1.1387	32.02	49.89	50.11		
boulevard	−0.0012	0.0380	32.75	0.0037	2.6333	7.18	47.83	52.17		
broad	−0.0063	0.1948	2.93	0.0038	2.1666	11.46	48.22	51.78		
cancel	0.0010	0.0297	66.09	0.0037	1.9699	13.95	48.33	51.67	$\ominus$	$\ominus$
cash	0.0020	0.0609	79.54	0.0044	0.5187	59.53	49.10	50.90		
challeng	−0.0054	0.1650	5.85	0.0039	1.8592	15.58	48.41	51.59	$\ominus$	$\ominus$
channel	0.0005	0.0158	60.24	0.0037	2.7430	6.44	48.90	51.10		
collater	−0.0010	0.0315	37.43	0.0039	2.4042	9.03	48.64	51.36		
communiti	−0.0011	0.0330	36.26	0.0039	2.0075	13.43	48.45	51.55	$\oplus$	
complex	−0.0013	0.0399	31.58	0.0038	2.9757	5.10	48.81	51.19	$\ominus$	
complianc	−0.0029	0.0901	15.21	0.0039	1.3746	25.30	48.29	51.71	$\oplus$	
compris	0.0041	0.1254	88.89	0.0037	1.8776	15.30	47.88	52.12		
consumm	0.0067	0.2045	94.16	0.0040	1.8885	15.13	48.43	51.57	$\oplus$	
continu	−0.0039	0.1197	8.78	0.0042	0.4519	63.64	48.94	51.06	$\oplus$	
contractor	0.0014	0.0438	73.10	0.0037	2.5583	7.74	48.84	51.16		
convert	−0.0001	0.0034	49.71	0.0038	1.5197	21.88	48.48	51.52		
core	−0.0014	0.0429	28.66	0.0039	1.9602	14.08	49.37	50.63		
customari	0.0011	0.0336	69.60	0.0039	1.9746	13.88	48.69	51.31		
david	−0.0038	0.1161	9.36	0.0037	2.2910	10.12	47.95	52.05		
deal	−0.0034	0.1041	11.70	0.0038	2.1553	11.59	48.28	51.72	$\oplus$	
decis	−0.0006	0.0183	42.11	0.0037	1.3938	24.81	48.41	51.59		
declin	−0.0204	0.6285	0.01	0.0045	1.4445	23.59	48.65	51.35	$\ominus$	$\ominus$
decreas	−0.0017	0.0533	24.57	0.0047	1.0913	33.58	48.80	51.20	$\ominus$	
delay	−0.0091	0.2785	2.34	0.0038	1.6758	18.72	47.65	52.35	$\ominus$	$\ominus$
depreci	−0.0051	0.1580	7.02	0.0052	1.4649	23.11	48.59	51.41	$\ominus$	
dial	0.0000	0.0009	52.64	0.0040	2.0318	13.11	49.78	50.22		
difficult	−0.0002	0.0061	48.54	0.0039	2.1912	11.18	47.88	52.12	$\ominus$	$\ominus$
digit	−0.0025	0.0765	19.30	0.0040	2.8527	5.77	49.04	50.96		
electron	0.0010	0.0322	68.43	0.0037	1.7849	16.78	49.23	50.77		
enabl	0.0023	0.0720	82.46	0.0038	1.7140	18.01	49.35	50.65	$\oplus$	$\oplus$
enact	−0.0027	0.0821	17.55	0.0037	2.7272	6.54	48.03	51.97		
ensur	−0.0012	0.0372	33.34	0.0038	2.0278	13.16	47.84	52.16	$\oplus$	
equal	−0.0008	0.0249	38.02	0.0042	1.3021	27.20	47.97	52.03	$\oplus$	
european	0.0019	0.0571	77.20	0.0038	2.4678	8.48	48.53	51.47		
exceed	0.0022	0.0676	81.88	0.0039	1.3360	26.29	49.28	50.72		
excel	0.0044	0.1345	90.65	0.0038	2.5333	7.94	50.80	49.20	$\oplus$	$\oplus$

except	0.0045	0.1377	91.23	0.0043	0.5213	59.38	49.04	50.96	$\ominus$	$\oplus$
expens	0.0014	0.0429	72.52	0.0050	0.6084	54.42	48.94	51.06	$\ominus$	
experienc	−0.0117	0.3585	1.76	0.0038	2.1062	12.17	47.93	52.07		
exposur	−0.0024	0.0725	19.89	0.0038	2.2922	10.10	47.83	52.17		
extens	0.0017	0.0527	76.03	0.0038	1.6726	18.78	48.40	51.60		
extern	−0.0004	0.0135	45.03	0.0037	2.5724	7.64	47.83	52.17		
facil	0.0106	0.3270	97.67	0.0039	1.0418	35.28	49.29	50.71		
favor	0.0073	0.2234	94.74	0.0040	1.3686	25.45	49.71	50.29	$\oplus$	$\oplus$
fit	−0.0004	0.0130	45.62	0.0038	2.8871	5.57	48.93	51.07	$\oplus$	
forecast	−0.0002	0.0054	49.13	0.0038	1.9290	14.53	49.00	51.00		
four	0.0001	0.0031	54.98	0.0037	1.5259	21.74	49.02	50.98		
gift	−0.0022	0.0677	22.23	0.0037	2.9462	5.25	47.95	52.05	$\oplus$	
goodwil	−0.0007	0.0229	39.19	0.0039	2.1187	12.02	48.85	51.15		
grade	−0.0013	0.0402	30.41	0.0038	2.8257	5.93	49.26	50.74		
grew	0.0014	0.0427	71.93	0.0042	2.4272	8.83	50.78	49.22		
hand	0.0004	0.0108	57.90	0.0038	1.9460	14.28	49.08	50.92	$\oplus$	
harbor	0.0012	0.0364	70.77	0.0038	1.6846	18.55	49.53	50.47		
highlight	0.0024	0.0725	83.05	0.0039	2.2062	11.01	50.01	49.99	$\oplus$	
holiday	−0.0015	0.0467	25.15	0.0037	2.8502	5.78	47.65	52.35		
howev	−0.0015	0.0447	26.91	0.0039	1.1339	32.18	48.49	51.51		
identifi	0.0006	0.0180	61.41	0.0040	1.0003	36.78	49.23	50.77		
impact	−0.0041	0.1258	7.61	0.0046	0.9215	39.79	48.62	51.38		
improv	0.0325	1.0000	100.00	0.0045	0.9870	37.27	49.70	50.30	$\oplus$	$\oplus$
includ	0.0010	0.0321	67.84	0.0042	0.0093	99.07	48.61	51.39		
increas	0.0113	0.3466	98.25	0.0051	0.5023	60.51	49.16	50.84		
inher	0.0001	0.0020	53.81	0.0039	2.3846	9.21	49.63	50.37		
injunct	0.0019	0.0578	77.78	0.0037	2.7181	6.60	47.96	52.04	$\ominus$	$\ominus$
interim	−0.0028	0.0867	16.38	0.0037	2.4850	8.33	47.96	52.04		
introduct	−0.0035	0.1089	10.53	0.0038	2.8800	5.61	48.11	51.89		
larger	−0.0001	0.0027	50.30	0.0038	2.8339	5.88	47.64	52.36		
led	0.0003	0.0104	57.31	0.0038	2.5818	7.56	49.48	50.52		
leverag	0.0026	0.0811	84.80	0.0039	1.8755	15.33	49.49	50.51		
like	−0.0005	0.0160	42.70	0.0040	1.4894	22.55	48.41	51.59	$\oplus$	
live	0.0032	0.0975	86.55	0.0038	1.4484	23.49	49.55	50.45	$\oplus$	
local	0.0019	0.0580	78.37	0.0039	1.4601	23.22	48.62	51.38		
longer	−0.0014	0.0445	28.08	0.0037	1.7377	17.59	47.96	52.04		
lower	−0.0138	0.4233	1.17	0.0047	1.2995	27.27	48.72	51.28	$\ominus$	
mandatori	0.0017	0.0515	75.44	0.0037	2.7091	6.66	48.77	51.23		
membership	0.0020	0.0628	80.12	0.0038	2.6589	7.00	49.37	50.63		
met	−0.0005	0.0159	43.28	0.0037	2.3760	9.29	48.19	51.81		
middl	0.0046	0.1400	91.82	0.0037	2.9644	5.16	49.15	50.85		
million	0.0010	0.0311	67.26	0.0056	0.5051	60.34	49.14	50.86		
minus	0.0040	0.1220	88.31	0.0038	2.7399	6.46	48.30	51.70		
negat	−0.0162	0.4965	0.59	0.0040	1.6080	20.03	47.85	52.15	$\ominus$	$\ominus$
noth	−0.0021	0.0643	23.40	0.0041	2.0435	12.96	48.13	51.87		
now	−0.0054	0.1652	5.27	0.0040	1.3043	27.14	48.80	51.20		
obtain	−0.0023	0.0720	21.06	0.0039	1.2424	28.87	48.20	51.80	$\oplus$	
offer	−0.0052	0.1586	6.44	0.0045	0.9082	40.33	48.76	51.24	$\oplus$	
ohio	0.0039	0.1196	87.72	0.0037	2.6206	7.28	49.88	50.12		
ordinari	0.0015	0.0449	74.27	0.0038	2.0600	12.75	48.19	51.81		
own	0.0042	0.1298	90.06	0.0039	1.0652	34.46	48.84	51.16		
pass	−0.0004	0.0118	46.20	0.0037	2.1973	11.11	48.42	51.58	$\oplus$	
payrol	0.0049	0.1498	92.40	0.0037	2.7043	6.69	49.32	50.68		
percentag	0.0021	0.0633	80.71	0.0039	1.2674	28.16	49.15	50.85		
placement	−0.0015	0.0460	26.32	0.0037	2.8314	5.89	47.94	52.06		
pleas	0.0028	0.0857	85.97	0.0040	1.0498	35.00	49.80	50.20	$\oplus$	$\oplus$
posit	0.0042	0.1296	89.48	0.0040	1.8227	16.16	49.58	50.42	$\oplus$	$\oplus$
predecessor	−0.0032	0.0986	13.46	0.0037	2.5207	8.04	47.86	52.14		
premium	0.0013	0.0396	71.35	0.0039	1.5912	20.37	48.77	51.23	$\oplus$	
pressur	−0.0055	0.1682	4.68	0.0038	2.3621	9.42	48.96	51.04		
print	0.0000	0.0004	52.05	0.0038	2.3379	9.65	48.60	51.40		
prior	0.0025	0.0770	83.63	0.0041	0.6143	54.10	48.70	51.30		
program	0.0014	0.0446	73.69	0.0040	1.0781	34.02	49.48	50.52		
project	−0.0055	0.1685	4.10	0.0041	0.9976	36.88	48.84	51.16		
provid	0.0006	0.0175	60.82	0.0043	0.3127	73.14	48.63	51.37	$\oplus$	
rais	0.0160	0.4932	99.42	0.0038	2.2016	11.06	51.17	48.83	$\ominus$	
rang	−0.0003	0.0096	47.37	0.0039	1.2950	27.39	48.99	51.01		

rapid	−0.0022	0.0691	21.64	0.0038	2.8107	6.02	48.44	51.56		
raw	−0.0004	0.0137	43.86	0.0040	2.3531	9.51	50.38	49.62		
record	0.0002	0.0076	56.73	0.0040	0.7359	47.91	49.05	50.95		
reduc	−0.0025	0.0773	18.72	0.0040	1.0160	36.20	48.55	51.45		
referenc	−0.0003	0.0086	47.96	0.0037	2.4892	8.30	47.60	52.40		
reflect	0.0015	0.0466	74.86	0.0043	0.6936	49.98	49.02	50.98		
regular	−0.0007	0.0218	40.94	0.0038	1.8729	15.37	47.91	52.09		
remain	−0.0032	0.0978	14.04	0.0038	0.7368	47.86	48.93	51.07		
reorgan	−0.0033	0.1028	12.87	0.0037	1.9715	13.92	48.00	52.00		
repurchas	0.0074	0.2278	95.33	0.0039	1.4710	22.97	50.03	49.97		
reput	0.0009	0.0279	65.50	0.0037	2.6938	6.76	48.21	51.79	$\oplus$	
respond	−0.0018	0.0559	23.98	0.0037	2.6823	6.84	48.37	51.63		
retain	0.0011	0.0325	69.01	0.0037	1.3337	26.35	49.05	50.95		
review	−0.0029	0.0900	15.79	0.0038	1.2681	28.14	48.38	51.62		
rise	−0.0021	0.0654	22.81	0.0038	1.9737	13.89	48.58	51.42		
secur	0.0027	0.0835	85.39	0.0041	1.0411	35.31	48.58	51.42	$\oplus$	
shift	−0.0031	0.0957	14.62	0.0038	2.9439	5.27	48.31	51.69		
short	−0.0007	0.0224	39.77	0.0037	2.1529	11.61	47.75	52.25	$\ominus$	
situat	−0.0026	0.0798	18.13	0.0037	2.7001	6.72	47.79	52.21		
six	0.0008	0.0241	64.33	0.0038	1.4168	24.25	49.08	50.92		
skill	−0.0013	0.0401	31.00	0.0037	2.9910	5.02	47.75	52.25	$\oplus$	
spend	−0.0008	0.0238	38.60	0.0039	2.1316	11.86	49.57	50.43		
staff	−0.0013	0.0394	32.17	0.0037	2.6853	6.82	47.58	52.42		
statist	0.0004	0.0115	58.48	0.0038	2.2625	10.41	49.25	50.75		
strateg	0.0026	0.0801	84.22	0.0038	1.5980	20.23	49.88	50.12		
strong	0.0144	0.4431	98.84	0.0045	1.2582	28.42	50.32	49.68		$\oplus$
stronger	0.0080	0.2460	96.50	0.0039	2.9114	5.44	50.58	49.42		$\oplus$
subordin	0.0000	0.0013	53.22	0.0038	2.2765	10.26	48.13	51.87		
suffici	−0.0015	0.0447	27.49	0.0039	1.6211	19.77	48.59	51.41	$\oplus$	
swap	−0.0011	0.0340	35.68	0.0039	2.3349	9.68	48.22	51.78		
take	−0.0004	0.0136	44.45	0.0040	1.2203	29.51	48.62	51.38		
tangibl	−0.0035	0.1085	11.12	0.0040	2.5342	7.93	48.54	51.46		
third	−0.0034	0.1031	12.29	0.0039	0.9116	40.19	48.58	51.42		
treasuri	0.0005	0.0155	59.07	0.0039	1.7271	17.78	48.93	51.07		
treat	0.0011	0.0343	70.18	0.0038	1.8629	15.52	48.89	51.11	$\oplus$	
undertak	0.0002	0.0061	56.15	0.0040	1.1176	32.70	48.52	51.48		
unfavor	−0.0011	0.0348	34.51	0.0039	2.6569	7.02	48.47	51.53	$\ominus$	$\ominus$
univers	−0.0027	0.0829	16.96	0.0038	2.6309	7.20	47.86	52.14		
unlaw	−0.0013	0.0412	29.24	0.0042	2.5861	7.53	47.63	52.37	$\ominus$	$\ominus$
usa	−0.0013	0.0403	29.83	0.0037	2.3757	9.30	48.12	51.88		
vacat	0.0076	0.2323	95.91	0.0037	2.8868	5.58	49.72	50.28		
various	−0.0011	0.0340	35.09	0.0038	1.3206	26.70	48.01	51.99		
venu	0.0020	0.0608	78.95	0.0037	2.9128	5.43	48.40	51.60		
volum	−0.0015	0.0463	25.74	0.0042	1.6508	19.19	48.87	51.13		
waiver	0.0095	0.2925	97.08	0.0039	1.8754	15.33	48.33	51.67		
weak	−0.0039	0.1209	8.19	0.0038	2.4553	8.58	48.35	51.65	$\ominus$	$\ominus$
world	0.0007	0.0207	62.58	0.0038	1.6043	20.10	50.24	49.76		
yet	−0.0012	0.0368	33.92	0.0037	2.0849	12.43	48.72	51.28		

F.3. Overlapping polarity expressions in movie reviews and financial filings

Table 7 reports terms that were selected by our procedure from both movie reviews and financial disclosures. In addition, we provide the ratio of documents with a positive or negative label. The symbol "✓" indicates terms that show an equal coefficient sign in both word lists, whereas "✗" indicates a disagreement regarding the word connotation.

Table 7.: Polarity terms that are statistically relevant in both movie reviews and financial filings.

	Panel I: Movie Reviews			Panel II: Financial Filings			
**Word Stem**	**Coef.**	**Positive Doc. (%)**	**Negative Doc. (%)**	**Coef.**	**Positive Doc. (%)**	**Negative Doc. (%)**	**Equal Sign (i. e. Connotation)**
abil	0.0135	0.66	0.34	0.0001	0.49	0.51	✓
actual	−0.0229	0.54	0.46	0.0007	0.49	0.51	✗
allow	0.0148	0.66	0.34	0.0022	0.49	0.51	✓
although	0.0254	0.67	0.33	−0.0036	0.48	0.52	✗
alway	0.0334	0.65	0.35	0.0010	0.50	0.50	✓
appear	−0.0186	0.57	0.43	0.0000	0.49	0.51	✓
becam	0.0016	0.61	0.39	0.0008	0.49	0.51	✓
better	−0.0092	0.55	0.45	0.0007	0.49	0.51	✗
challeng	0.0181	0.68	0.32	−0.0054	0.48	0.52	✗
complex	0.0304	0.78	0.22	−0.0013	0.49	0.51	✗
deal	0.0059	0.63	0.37	−0.0034	0.48	0.52	✗
decis	−0.0064	0.56	0.44	−0.0006	0.48	0.52	✓
equal	0.0115	0.71	0.29	−0.0008	0.48	0.52	✗
excel	0.0572	0.65	0.35	0.0044	0.51	0.49	✓
fit	0.0080	0.63	0.37	−0.0004	0.49	0.51	✗
howev	0.0210	0.65	0.35	−0.0015	0.48	0.52	✗
impact	0.0048	0.71	0.29	−0.0041	0.49	0.51	✗
includ	0.0019	0.61	0.39	0.0010	0.49	0.51	✓
like	−0.0331	0.57	0.43	−0.0005	0.48	0.52	✓
local	0.0079	0.63	0.37	0.0019	0.49	0.51	✓
noth	−0.0018	0.56	0.44	−0.0021	0.48	0.52	✓
pass	0.0004	0.55	0.45	−0.0004	0.48	0.52	✗
posit	0.0013	0.60	0.40	0.0042	0.50	0.50	✓
project	−0.0157	0.55	0.45	−0.0055	0.49	0.51	✓
provid	−0.0053	0.60	0.40	0.0006	0.49	0.51	✗
rais	0.0079	0.65	0.35	0.0160	0.51	0.49	✓
rang	0.0008	0.61	0.39	−0.0003	0.49	0.51	✗
reflect	0.0198	0.62	0.38	0.0015	0.49	0.51	✓
remain	0.0029	0.64	0.36	−0.0032	0.49	0.51	✗
review	−0.0741	0.51	0.49	−0.0029	0.48	0.52	✓
short	0.0069	0.62	0.38	−0.0007	0.48	0.52	✗
spend	−0.0124	0.56	0.44	−0.0008	0.50	0.50	✓
strong	0.0140	0.69	0.31	0.0144	0.50	0.50	✓
third	0.0124	0.65	0.35	−0.0034	0.49	0.51	✗
treat	0.0075	0.60	0.40	0.0011	0.49	0.51	✓
univers	−0.0005	0.59	0.41	−0.0027	0.48	0.52	✓
world	0.0123	0.57	0.43	0.0007	0.50	0.50	✓
yet	0.0339	0.67	0.33	−0.0012	0.49	0.51	✗

Appendix G: List of polarity bigrams

In the following section, we report the complete list of word stem bigrams that result from our statistical procedure of extracting polarity expressions.

G.1. Study I: Movie reviews

Table 8 reports word stem bigrams that convey positive or negative sentiment in movie reviews. In addition, we provide the ratio of documents with a positive or negative label.

Table 8.: Empirical results of opinionated word stem bigrams in movie reviews.

Word Stem	Coef.	Positive Doc. (%)	Negative Doc. (%)
academi award	0.0528	0.80	0.20
accept kid	−0.0256	0.43	0.57
accept older	−0.0349	0.45	0.55
accept teenagers	−0.0253	0.46	0.54
act like	−0.0235	0.40	0.60
action film	−0.0216	0.46	0.54
action scene	−0.0183	0.35	0.65
age gave	−0.0005	0.51	0.49
allow us	0.0211	0.86	0.14
along way	0.0023	0.64	0.36
alway seem	0.0063	0.56	0.44
answer question	0.0053	0.65	0.35
anyth els	0.0087	0.62	0.38
april opinion	−0.0051	0.61	0.39
ask question	0.0055	0.56	0.44
aspect film	0.0013	0.68	0.32
audienc will	−0.0015	0.53	0.47
award nomin	0.0227	0.85	0.15
award one	0.0240	0.80	0.20
back home	0.0030	0.58	0.42
bad film	−0.0303	0.39	0.61
bad guy	−0.0109	0.50	0.50
bad languag	0.0005	0.63	0.37
bad movi	−0.0640	0.32	0.68
bad news	−0.0218	0.38	0.62
base book	0.0063	0.61	0.39
base novel	0.0298	0.66	0.34
base play	0.0041	0.68	0.32
beauti film	0.0178	0.75	0.25
becom one	0.0132	0.67	0.33
best actor	0.0276	0.82	0.18
best film	0.0817	0.91	0.09
best known	0.0263	0.77	0.23
best line	0.0147	0.63	0.38
best perform	0.0234	0.86	0.14
best thing	−0.0038	0.50	0.50
bever hill	−0.0315	0.49	0.51
black white	0.0056	0.69	0.31
bond film	0.0048	0.61	0.39
brief nuditi	−0.0080	0.56	0.44
bring back	0.0156	0.63	0.37
bruce willi	−0.0109	0.53	0.47

camera work	0.0196	0.73	0.27
can easili	0.0083	0.68	0.32
can go	−0.0136	0.39	0.61
can said	−0.0154	0.28	0.72
can say	−0.0161	0.45	0.55
can see	0.0076	0.66	0.34
can take	0.0074	0.66	0.34
cant even	−0.0026	0.47	0.53
cant help	0.0035	0.61	0.39
chang mind	−0.0017	0.45	0.55
charact film	−0.0237	0.48	0.52
charact one	0.0043	0.73	0.27
charact play	0.0017	0.61	0.39
charact studi	0.0411	0.81	0.19
classif mpaa	0.0024	0.90	0.10
close credit	−0.0069	0.54	0.46
come close	−0.0129	0.53	0.47
come togeth	0.0042	0.66	0.34
credit roll	0.0037	0.71	0.29
decid take	0.0001	0.63	0.37
denni schwartz	−0.0890	0.41	0.59
didnt know	−0.0071	0.44	0.56
director peter	−0.0077	0.50	0.50
distributor miramax	0.0097	0.85	0.15
doesnt even	−0.0297	0.40	0.60
doesnt get	−0.0043	0.44	0.56
doesnt make	−0.0221	0.44	0.56
doesnt realli	0.0003	0.64	0.36
doesnt seem	−0.0034	0.55	0.45
doesnt want	0.0153	0.63	0.37
doesnt work	−0.0109	0.35	0.65
dont know	0.0072	0.56	0.44
dont think	−0.0070	0.48	0.52
drug dealer	−0.0003	0.49	0.51
drug usag	−0.0094	0.55	0.45
dysfunct famili	0.0022	0.69	0.31
earli film	0.0074	0.67	0.33
earli s	0.0265	0.74	0.26
edg seat	0.0566	0.88	0.12
en xrtsourceid	−0.0007	0.29	0.71
end movi	−0.0069	0.56	0.44
enough make	−0.0023	0.52	0.48
ensembl cast	0.0276	0.76	0.24
entertain film	0.0021	0.59	0.41
entertain valu	−0.0065	0.47	0.53
entir life	0.0065	0.73	0.27
entir movi	−0.0128	0.43	0.57
even better	0.0249	0.85	0.15
even get	−0.0160	0.41	0.59
even though	0.0529	0.64	0.36
even wors	−0.0496	0.28	0.72
ever made	0.0239	0.75	0.25
everi day	0.0082	0.69	0.31
everyon els	−0.0121	0.50	0.50
facial express	0.0107	0.73	0.27

fail attempt	−0.0201	0.33	0.67
fair amount	0.0016	0.63	0.37
far away	0.0041	0.67	0.33
fast rate	0.0107	1.00	0.00
favorit part	0.0136	0.73	0.27
fbi agent	−0.0003	0.44	0.56
februari opinion	−0.0096	0.53	0.47
fifteen minut	−0.0183	0.48	0.52
film also	0.0145	0.67	0.33
film becom	−0.0006	0.49	0.51
film can	0.0074	0.51	0.49
film certain	0.0042	0.57	0.43
film critic	0.0064	0.63	0.37
film ever	0.0027	0.69	0.31
film feel	−0.0034	0.42	0.58
film festiv	0.0173	0.74	0.26
film first	0.0095	0.56	0.44
film get	−0.0062	0.40	0.60
film go	−0.0010	0.40	0.60
film goe	0.0073	0.59	0.41
film just	−0.0321	0.38	0.62
film lack	−0.0307	0.26	0.74
film like	−0.0068	0.56	0.44
film littl	−0.0230	0.48	0.52
film look	−0.0433	0.34	0.66
film might	−0.0135	0.41	0.59
film move	0.0027	0.60	0.40
film much	0.0038	0.62	0.38
film noir	0.0013	0.62	0.38
film one	0.0035	0.56	0.44
film open	0.0008	0.55	0.45
film review	−0.0042	0.46	0.54
film s	0.0091	0.71	0.29
film seem	−0.0231	0.37	0.63
film sinc	0.0031	0.71	0.29
film tri	−0.0162	0.41	0.59
film use	−0.0004	0.44	0.56
film without	0.0059	0.62	0.38
film year	0.0132	0.61	0.39
find film	−0.0016	0.43	0.57
fine job	0.0026	0.69	0.31
fine kid	0.0341	0.75	0.26
fine line	0.0101	0.73	0.27
fine teenag	0.0087	0.70	0.30
fine teenagers	0.0234	0.74	0.26
first featur	0.0079	0.62	0.38
first hour	−0.0116	0.53	0.47
first part	0.0062	0.68	0.32
first time	0.0047	0.59	0.41
five minut	−0.0014	0.44	0.56
fun watch	−0.0050	0.43	0.58
get away	−0.0184	0.44	0.56
get caught	−0.0030	0.58	0.42
get involv	0.0033	0.53	0.47
get see	−0.0094	0.46	0.54

give film	0.0125	0.56	0.44
give movi	−0.0038	0.47	0.53
give must	0.0426	0.78	0.22
give one	0.0034	0.65	0.35
good evil	0.0082	0.65	0.35
good film	−0.0024	0.53	0.47
good idea	−0.0003	0.46	0.54
good movi	−0.0184	0.49	0.51
good thing	−0.0110	0.51	0.49
good time	0.0002	0.64	0.36
great film	0.0292	0.73	0.27
half film	0.0013	0.67	0.33
half hour	−0.0103	0.52	0.48
hard believ	−0.0197	0.32	0.68
hes just	−0.0093	0.51	0.49
high energi	0.0022	0.77	0.23
high point	−0.0008	0.54	0.46
high school	−0.0033	0.55	0.45
hollywood film	0.0027	0.59	0.41
horror film	−0.0130	0.41	0.59
horror movi	−0.0171	0.40	0.60
hour half	−0.0371	0.39	0.61
human be	0.0041	0.70	0.30
im sure	−0.0234	0.46	0.54
ingmar bergman	0.0108	0.67	0.33
interest charact	−0.0117	0.40	0.60
introduc us	−0.0018	0.61	0.39
isnt just	0.0020	0.64	0.36
isnt much	−0.0459	0.40	0.60
june opinion	−0.0071	0.54	0.46
jurass park	0.0036	0.60	0.40
just doesnt	−0.0148	0.41	0.59
just like	0.0051	0.51	0.49
just rate	−0.0088	0.58	0.42
keep audienc	0.0005	0.81	0.19
keep film	0.0117	0.63	0.37
kind movi	−0.0073	0.49	0.51
languag sexual	−0.0023	0.57	0.43
last act	0.0008	0.72	0.28
last seen	−0.0017	0.57	0.43
lead charact	0.0053	0.65	0.35
least one	0.0038	0.64	0.36
leav peopl	0.0007	0.70	0.30
leav theater	0.0045	0.71	0.29
length rate	0.0029	0.74	0.26
let go	0.0153	0.62	0.38
life one	0.0198	0.74	0.26
life take	0.0026	0.72	0.28
like see	0.0179	0.70	0.30
limit run	0.0404	0.88	0.12
line film	−0.0123	0.38	0.62
live life	0.0216	0.75	0.25
long enough	−0.0003	0.46	0.54
long rate	−0.0210	0.41	0.59
long time	0.0051	0.67	0.33

look back	0.0035	0.72	0.28
look good	−0.0243	0.38	0.62
look like	−0.0309	0.53	0.47
lot time	−0.0068	0.51	0.49
love film	0.0138	0.63	0.38
love interest	−0.0216	0.38	0.63
love life	0.0275	0.80	0.20
love one	0.0190	0.78	0.22
love stori	0.0076	0.69	0.31
made film	0.0036	0.53	0.47
make feel	0.0107	0.68	0.32
make film	0.0036	0.56	0.44
make love	0.0024	0.61	0.39
make mistak	−0.0028	0.54	0.46
make money	0.0016	0.56	0.44
make one	0.0003	0.60	0.40
make sens	−0.0249	0.42	0.58
make sure	−0.0256	0.45	0.55
make us	0.0046	0.67	0.33
make way	0.0064	0.73	0.27
man woman	0.0300	0.83	0.17
mani charact	0.0015	0.67	0.33
mani differ	−0.0078	0.47	0.53
mani film	0.0227	0.73	0.27
mani way	0.0402	0.79	0.21
martin scorses	0.0298	0.73	0.27
matur theme	0.0430	0.82	0.18
may find	−0.0113	0.57	0.43
may opinion	−0.0004	0.50	0.50
might expect	0.0065	0.72	0.28
might seem	0.0038	0.65	0.35
might well	−0.0444	0.36	0.64
million dollar	−0.0176	0.43	0.57
minut film	−0.0102	0.49	0.51
minut run	−0.0086	0.66	0.34
miramax films	0.0155	0.86	0.14
moment film	−0.0008	0.55	0.45
money total	−0.0231	0.61	0.39
motion pictur	0.0422	0.71	0.29
motion picture	0.0001	0.79	0.21
movi can	−0.0064	0.55	0.45
movi doesnt	−0.0146	0.41	0.59
movi featur	−0.0144	0.44	0.56
movi give	−0.0061	0.56	0.44
movi isnt	−0.0035	0.57	0.43
movi just	−0.0131	0.37	0.63
movi made	−0.0169	0.42	0.58
movi never	−0.0146	0.53	0.47
movi run	−0.0099	0.48	0.52
movi year	−0.0092	0.53	0.47
much better	−0.0249	0.48	0.52
much fun	−0.0055	0.51	0.49
much like	0.0028	0.59	0.41
music number	0.0070	0.66	0.34
never quit	0.0028	0.67	0.33

never seem	−0.0065	0.53	0.47
never seen	0.0028	0.67	0.33
nineti minut	−0.0207	0.46	0.54
noir film	0.0170	0.55	0.45
nuditi profan	−0.0063	0.63	0.37
old enough	0.0081	0.79	0.21
on day	−0.0006	0.60	0.40
one anoth	0.0044	0.60	0.40
one best	0.0804	0.85	0.15
one better	0.0047	0.71	0.29
one big	0.0031	0.63	0.37
one charact	−0.0021	0.53	0.47
one day	0.0101	0.66	0.34
one get	−0.0019	0.56	0.44
one man	0.0080	0.82	0.18
one night	−0.0153	0.50	0.50
one person	−0.0007	0.58	0.42
one thing	0.0067	0.56	0.44
one two	−0.0024	0.57	0.43
one worst	−0.0288	0.58	0.42
one year	0.0170	0.72	0.28
open scene	0.0004	0.64	0.36
oscar nomin	0.0310	0.89	0.11
pain unbear	0.0000	0.61	0.39
part movi	−0.0179	0.50	0.50
part stori	0.0062	0.66	0.34
perhap best	0.0033	0.71	0.29
pg adult	−0.0157	0.45	0.55
pg profan	−0.0132	0.52	0.48
pg sexual	0.0065	0.70	0.30
pg violenc	−0.0056	0.45	0.55
picture review	−0.0064	0.61	0.39
piec work	0.0103	0.73	0.27
play charact	0.0000	0.66	0.34
play game	0.0172	0.73	0.27
play like	−0.0160	0.49	0.51
press kit	0.0094	0.75	0.25
pretti much	−0.0070	0.41	0.59
previous film	0.0090	0.76	0.24
problem film	−0.0130	0.36	0.64
product design	0.0042	0.62	0.38
profan sexual	−0.0025	0.55	0.45
profan violenc	−0.0240	0.54	0.46
pulp fiction	0.0194	0.63	0.37
punch line	−0.0179	0.50	0.50
put togeth	−0.0278	0.50	0.50
quentin tarantino	0.0019	0.61	0.39
r sexual	0.0075	0.71	0.29
r violenc	−0.0202	0.56	0.44
rate pg	−0.0207	0.58	0.42
rate r	−0.0052	0.58	0.42
real life	0.0400	0.76	0.24
reason see	−0.0125	0.45	0.55
recommend film	0.0197	0.70	0.30
recommend give	−0.0158	0.39	0.61

recommend movi	0.0015	0.64	0.36
recommend pictur	0.0056	0.75	0.25
remind us	0.0179	0.73	0.27
renshaw screen	−0.0038	0.54	0.46
rest cast	−0.0010	0.52	0.48
rest movi	−0.0055	0.47	0.53
result film	−0.0038	0.47	0.53
review denni	−0.0771	0.40	0.60
revolv around	−0.0019	0.47	0.53
robert duval	0.0109	0.67	0.33
romant comedi	−0.0118	0.59	0.41
run around	−0.0260	0.43	0.57
run fast	0.0582	1.00	0.00
run length	0.0452	0.77	0.23
run littl	0.0056	0.85	0.15
run long	−0.0520	0.26	0.74
san francisco	−0.0145	0.49	0.51
san jose	0.0036	0.75	0.25
sandra bullock	−0.0030	0.58	0.42
saturday night	−0.0286	0.35	0.65
say film	−0.0064	0.40	0.60
scale scott	−0.0016	0.54	0.46
schwartz xramrid	−0.0069	0.37	0.63
scienc fiction	0.0098	0.64	0.36
screen presenc	−0.0099	0.51	0.49
screenplay john	−0.0099	0.61	0.39
sean conneri	0.0056	0.66	0.34
second half	0.0006	0.71	0.29
see movi	−0.0213	0.53	0.47
seem like	−0.0145	0.47	0.53
seen film	0.0278	0.63	0.37
sens humor	0.0185	0.75	0.25
serial killer	−0.0134	0.51	0.49
sex scene	−0.0132	0.43	0.57
sexual innuendo	−0.0010	0.56	0.44
show one	−0.0035	0.53	0.47
sight gag	−0.0334	0.38	0.62
small part	0.0028	0.63	0.37
small town	0.0090	0.61	0.39
soap opera	−0.0126	0.50	0.50
soni pictur	0.0224	0.88	0.12
sound like	−0.0052	0.54	0.46
special effect	−0.0230	0.48	0.53
spend time	−0.0044	0.55	0.45
stanford univers	−0.0118	0.54	0.46
star war	0.0397	0.58	0.42
stori concern	−0.0020	0.47	0.53
stori line	−0.0145	0.41	0.59
stori told	0.0136	0.71	0.29
stori two	0.0020	0.67	0.33
stori unfold	0.0342	0.80	0.20
strong perform	0.0147	0.77	0.23
subject matter	0.0225	0.69	0.31
support cast	0.0190	0.71	0.29
support player	0.0148	0.75	0.25

support role	0.0035	0.77	0.23
take place	0.0182	0.57	0.43
take us	0.0133	0.66	0.34
teenagers film	0.0177	0.71	0.29
televis seri	−0.0069	0.49	0.51
tell us	0.0017	0.58	0.42
ten minut	−0.0173	0.37	0.63
ten year	−0.0010	0.59	0.41
theatric aspect	0.0054	0.77	0.23
theme run	0.0062	0.59	0.41
theme theatric	0.0002	0.84	0.16
there much	−0.0212	0.46	0.54
there someth	0.0032	0.67	0.33
thing can	−0.0024	0.38	0.62
thing film	−0.0115	0.31	0.69
thing get	−0.0077	0.47	0.53
thing go	−0.0094	0.51	0.49
thing like	−0.0257	0.37	0.63
though film	0.0167	0.64	0.36
time film	0.0021	0.66	0.34
titl charact	0.0132	0.75	0.25
toward end	0.0048	0.72	0.28
tri hard	−0.0330	0.39	0.61
true love	0.0001	0.67	0.33
true stori	0.0138	0.66	0.34
turn one	0.0077	0.77	0.23
tv show	−0.0084	0.52	0.48
twentieth centuri	0.0093	0.67	0.33
twist turn	0.0096	0.73	0.27
two charact	0.0085	0.70	0.30
two peopl	0.0023	0.68	0.32
two year	0.0049	0.58	0.42
two young	0.0033	0.69	0.31
univers pictures	−0.0081	0.47	0.53
us avail	0.0116	0.70	0.30
via email	0.0084	0.62	0.38
violenc profan	−0.0020	0.61	0.39
want know	0.0087	0.59	0.41
war ii	0.0060	0.77	0.23
warner bros	−0.0207	0.39	0.61
wast money	−0.0297	0.60	0.40
wast time	−0.0605	0.28	0.72
well worth	0.0277	0.90	0.10
whole lot	−0.0108	0.50	0.50
wide run	−0.0269	0.55	0.45
will abl	0.0097	0.68	0.32
will get	0.0030	0.59	0.41
will go	0.0008	0.62	0.38
will leav	0.0315	0.74	0.26
will never	0.0143	0.65	0.35
will probabl	−0.0045	0.53	0.47
will take	0.0170	0.66	0.34
without ever	0.0288	0.87	0.13
wonder whether	−0.0022	0.54	0.46
woodi allen	0.0047	0.76	0.24

work hard	0.0073	0.64	0.36
work way	0.0106	0.61	0.39
work well	0.0160	0.66	0.34
world war	0.0135	0.71	0.29
worst film	−0.0023	0.58	0.42
written april	−0.0009	0.61	0.39
written direct	−0.0045	0.58	0.42
written june	0.0000	0.54	0.46
written march	0.0010	0.63	0.37
year ago	0.0052	0.64	0.36
year old	0.0233	0.65	0.35
yet anoth	−0.0047	0.52	0.48
young man	0.0206	0.65	0.35
young woman	0.0196	0.76	0.24
younger brother	−0.0057	0.48	0.52

G.2. Study II: Financial filings

Table 9 reports word stem bigrams that convey positive or negative sentiment in financial filings. In addition, we provide the ratio of documents with a positive or negative label.

Table 9.: Empirical results of opinionated word stem bigrams in financial filings.

Word Stem	Coef.	Positive Doc. (%)	Negative Doc. (%)
act section	−0.0017	0.49	0.51
affect busi	−0.0007	0.48	0.52
agre write	−0.0023	0.49	0.51
agreement case	−0.0042	0.48	0.52
author share	0.0005	0.49	0.51
base salari	0.0015	0.48	0.52
certif sign	−0.0001	0.50	0.50
compani corpor	0.0015	0.48	0.52
control common	0.0210	0.49	0.51
deem includ	0.0000	0.51	0.50
descript exhibit	−0.0006	0.49	0.51
elect board	0.0030	0.50	0.50
estim project	0.0013	0.49	0.51
file item	0.0015	0.49	0.51
forth articl	0.0240	0.49	0.51
inc compani	−0.0008	0.49	0.51
incom increas	0.0020	0.49	0.51
interest tax	−0.0028	0.48	0.52
issu outstand	0.0014	0.48	0.52
known unknown	0.0052	0.47	0.53
law shall	−0.0016	0.48	0.52
may effect	−0.0001	0.49	0.51
mean date	−0.0018	0.48	0.52
measur includ	−0.0005	0.48	0.52
million prior	0.0028	0.49	0.51
negat impact	−0.0112	0.49	0.51
new jersey	0.0048	0.49	0.51
north carolina	−0.0009	0.49	0.51
part thereof	−0.0002	0.49	0.51
perform observ	−0.0008	0.48	0.52
provis articl	−0.0025	0.48	0.52
quarter decreas	−0.0082	0.49	0.51
quarter increas	0.0043	0.49	0.51
receiv notic	−0.0033	0.48	0.52
releas statement	0.0022	0.47	0.53
requir applic	−0.0022	0.49	0.51
requir made	−0.0001	0.47	0.53
requir section	−0.0018	0.47	0.53
respect claim	−0.0017	0.48	0.52
result may	0.0000	0.48	0.52
right receiv	−0.0004	0.50	0.50
sale cost	0.0034	0.49	0.51
sale increas	0.0055	0.49	0.51
stock par	0.0033	0.48	0.52
supersed prior	0.0030	0.48	0.52
tax depreci	0.0000	0.49	0.51
unreason withheld	−0.0045	0.48	0.52

References

[1] Manning CD, Schütze H. Foundations of Statistical Natural Language Processing. Cambridge, MA: MIT Press; 1999.

[2] Loughran T, McDonald B. When Is a Liability Not a Liability? Textual Analysis, Dictionaries, and 10-Ks. Journal of Finance. 2011;66(1):35–65.

[3] Feinerer I, Hornik K, Meyer D. Text Mining Infrastructure in R. Journal of Statistical Software. 2008;25(5):1–54.

[4] Porter MF. An Algorithm for Suffix Stripping. Program: Electronic Library and Information Systems. 1980;14(3):130–137.

[5] Kiritchenko S, Zhu X, Mohammad SM. Sentiment Analysis of Short Informal Texts. Journal of Artificial Intelligence Research. 2014;50:723–762.

[6] Oliveira N, Cortez P, Areal N. Stock Market Sentiment Lexicon Acquisition Using Microblogging Data and Statistical Measures. Decision Support Systems. 2016;85(5):62–73.

[7] Salton G, Fox EA, Wu H. Extended Boolean Information Retrieval. Communications of the ACM. 1983;26(11):1022–1036.

[8] MacKinlay AC. Event Studies in Economics and Finance. Journal of Economic Literature. 1997;35(1):13–39.

[9] Konchitchki Y, O'Leary DE. Event Study Methodologies in Information Systems Research. International Journal of Accounting Information Systems. 2011;12(2):99–115.

[10] Jegadeesh N, Wu D. Word Power: A New Approach For Content Analysis. Journal of Financial Economics. 2013;110(3):712–729.

[11] Hastie T, Tibshirani R, Friedman JH. The Elements of Statistical Learning: Data Mining, Inference, and Prediction. 2nd ed. Springer Series in Statistics. New York: Springer; 2009.

[12] Manevitz LM, Yousef M. One-Class SVMs for Document Classification. Journal of Machine Learning Research. 2001;2(1):139–154.

[13] Tetlock PC. Giving Content to Investor Sentiment: The Role of Media in the Stock Market. Journal of Finance. 2007;62(3):1139–1168.

[14] Belloni A, Chernozhukov V. Least Squares After Model Selection in High-Dimensional Sparse Models. Bernoulli. 2013;19(2):521–547.

Figure captions

Fig 1. Kernel density estimation of word polarity scores from statistical inferences.
